# Aspirin versus low-molecular-weight heparin for venous thromboembolism prophylaxis in orthopaedic trauma patients: A patient-centered randomized controlled trial

**DOI:** 10.1371/journal.pone.0235628

**Published:** 2020-08-03

**Authors:** Bryce E. Haac, Nathan N. O'Hara, Theodore T. Manson, Gerard P. Slobogean, Renan C. Castillo, Robert V. O'Toole, Deborah M. Stein, on behalf of the ADAPT Investigators

**Affiliations:** 1 Department of Surgery, R A Cowley Shock Trauma Center, University of Maryland School of Medicine, Baltimore, Maryland, United States of America; 2 Department of Orthopaedics, R A Cowley Shock Trauma Center, University of Maryland School of Medicine, Baltimore, Maryland, United States of America; 3 Department of Health Policy and Management, Johns Hopkins University Bloomberg School of Public Health, Baltimore, Maryland, United States of America; Universite de Bretagne Occidentale, FRANCE

## Abstract

**Background:**

Emerging evidence suggests aspirin may be an effective venous thromboembolism (VTE) prophylaxis for orthopaedic trauma patients, with fewer bleeding complications. We used a patient-centered weighted composite outcome to globally evaluate aspirin versus low-molecular-weight heparin (LMWH) for VTE prevention in fracture patients.

**Methods:**

We conducted an open-label randomized clinical trial of adult patients admitted to an academic trauma center with an operative extremity fracture, or a pelvis or acetabular fracture. Patients were randomized to receive LMWH (enoxaparin 30-mg) twice daily (n = 164) or aspirin 81-mg twice daily (n = 165). The primary outcome was a composite endpoint of bleeding complications, deep surgical site infection, deep vein thrombosis, pulmonary embolism, and death within 90 days of injury. A Global Rank test and weighted time to event analysis were used to determine the probability of treatment superiority for LMWH, given a 9% patient preference margin for oral administration over skin injections.

**Results:**

Overall, 18 different combinations of outcomes were experienced by patients in the study. Ninety-nine patients in the aspirin group (59.9%) and 98 patients in the LMWH group (59.4%) were event-free within 90 days of injury. Using a Global Rank test, the LMWH had a 50.4% (95% CI, 47.7–53.2%, p = 0.73) probability of treatment superiority over aspirin. In the time to event analysis, LMWH had a 60.5% probability of treatment superiority over aspirin with considerable uncertainty (95% CI, 24.3–88.0%, p = 0.59).

**Conclusion:**

The findings of the Global Rank test suggest no evidence of superiority between LMWH or aspirin for VTE prevention in fracture patients. LMWH demonstrated a 60.5% VTE prevention benefit in the weighted time to event analysis. However, this difference did not reach statistical significance and was similar to the elicited patient preferences for aspirin.

## Introduction

Trauma is a well-described risk factor for venous thromboembolism (VTE) [1–8], but controversy exists regarding the optimal thromboprophylaxis regimen following orthopaedic trauma [9–11]. The Eastern Association for the Surgery of Trauma and the American College of Chest Physicians (ACCP) recommend low-molecular-weight heparin (LMWH) for VTE prophylaxis in trauma patients [11, 12]. However, many orthopaedic trauma surgeons prefer aspirin in light of recent studies suggesting aspirin may be an effective alternative in VTE prevention with a reduced likelihood of bleeding and wound complications [13–19]. As a result, the ACCP guidelines now include aspirin as an option for high-risk orthopaedic surgery patients [11]. To date, most randomized studies comparing these regimens have been conducted in the arthroplasty population. Considering that orthopaedic trauma patients often have multiple injuries that may increase VTE and bleeding risks, drawing inference from the arthroplasty literature is cautioned. Based on the lack of scientific support for various regimens in this population, the Orthopaedic Trauma Association Evidence-Based Quality Value and Safety Committee recently emphasized the need for standardized VTE prevention guidelines to improve care [20].

Like many treatment decisions, determining the optimal thromboprophylaxis regimen for a given patient requires clinicians to weigh the benefits and risks of the available treatment options. Clinical trials traditionally have a separate comparison between the study groups for each study outcome. The analysis technique used in this trial was based on the foundational work by Evans and Follman [21], where the study patient’s experiences are compared between the two treatment groups, allowing multiple outcomes to be counted for a given patient.

The clinical relevance of this patient-centered approach can be improved further using composite outcomes that incorporate patient preferences to weigh or rank the plausible outcomes. Techniques, such as a Global Rank test or time to event analysis with weighted, repeated events, can determine the probability of treatment superiority among two or more treatment options [21, 22]. The elicited patient preferences can also be used to determine the preferred treatments under various patient-important thresholds for treatment superiority.

We performed a randomized controlled trial to globally evaluate aspirin versus LMWH for VTE prophylaxis after orthopaedic trauma. In this study, we incorporated several innovative techniques in patient-centered research. Patient preference data was used to weigh the relative importance of the outcomes included our composite endpoint [23], which included bleeding complications, deep surgical site infections, deep vein thrombosis, pulmonary embolism, and death. The patient preference data were also used to set our threshold for a significant difference between the prophylaxis options under the consideration that patients preferred the route of administration for aspirin over the LWMH injections.

## Materials and methods

### Trial design

The A Different Approach to Preventing Thrombosis (ADAPT) trial was a single-center open-label prospective randomized controlled trial that compared the global benefit of aspirin versus LMWH for orthopaedic trauma patients within 90 days of injury. Global benefit being the synthesis of several patient-important outcomes. Patients were enrolled from January through November 2016 at an academic trauma center in Baltimore, Maryland. Patient follow-up was completed in April 2017. Ethics approval was obtained from the University of Maryland institutional review board (IRB) on September 12, 2015, and informed consent was obtained for all enrolled patients. The trial was registered in clinicaltrials.gov (NCT02774265) on May 17, 2016. Due to an administrative oversight, the trial was registered after patient enrolment was initiated, but prior to the completion of patient enrolment. The authors confirm that all ongoing and related trials for this drug have been registered.

### Patient selection

All adult trauma patients admitted with an operative extremity fracture proximal to the carpals or metatarsals, or any hip or acetabular fracture requiring VTE prophylaxis were included in the study. We excluded prisoners, pregnant patients, non-English-speaking patients, patients with an indication for therapeutic anticoagulation or high-dose aspirin (>81 mg daily), and patients receiving pre-existing VTE prophylaxis, therapeutic anticoagulation before admission, or patients that received more than two doses of VTE prophylaxis before consent. Patients with a VTE within the last six months, impaired creatinine clearance (≤30 mL/min), history of heparin-induced thrombocytopenia or aspirin or non-steroidal anti-inflammatory allergy, or another contraindication to receiving a study medication were also excluded. Eligible patients were approached by a member of the research team within 48 hours of admission to the study location and enrolled after obtaining written informed consent.

### Randomization

Patients were randomly assigned in a 1:1 ratio to VTE prophylaxis by LMWH or aspirin. Randomization was performed with REDCap, using blocks of six, by research staff at the time of enrollment. The study group allocation was not concealed to the patients, surgeons, or research staff.

### Study intervention and procedures

Patients allocated to the LMWH group received 30-mg doses of enoxaparin twice daily with allowance for dose adjustment based on body mass index or anti-factor Xa levels. Patients allocated to the aspirin group received 81-mg doses twice daily. Administration of aspirin was oral, rectal, or via any other form of enteral access. The off-label use of 81-mg aspirin for the indication of VTE prophylaxis was approved by the IRB of record. The duration of prophylaxis was based on hospital guidelines and was dependent on fracture location and weightbearing status. Mechanical prophylaxis with intermittent pneumatic compression devices was ordered as part of the clinical protocol for all inpatients. Study follow-up was conducted 90 days after fracture either at the patient’s clinic appointment or by phone. A chart review was conducted at that time to ensure that no events were missed in the patient review.

All prophylaxis doses were monitored daily during the index admission by the study’s research staff. Additionally, medication adherence was assessed at all clinical follow-up visits. Aspirin use by patients for reasons other than VTE prophylaxis was also tracked. Medication contamination and unplanned crossover were recorded.

### Study outcomes

The primary outcome was a composite that included bleeding complications, VTE, deep surgical site infections, and death occurring within 90 days after injury. Bleeding complications were defined as a ≥2 g/dL drop in hemoglobin within a 24-hour period after initiation of the study medication, blood transfusion, gastrointestinal bleeding, surgical site hematoma requiring reoperation, or other bleeding events requiring intervention or after initiation of the prophylaxis regimen. The administration of a blood transfusion was at the discretion of the treating clinician who ordered the transfusion. VTE events included pulmonary embolism (PE) defined as either massive, submassive, or clinically significant PE, and clinically significant deep vein thrombosis (DVT) occurring after initiation of the prophylaxis regimen. No screening of asymptomatic patients for VTE events was conducted. Massive and submassive PE were defined using the American Heart Association definitions [24]. Clinically significant PE was defined as symptomatic PE that did not fit massive or submassive criteria. Clinically significant DVT was defined as symptomatic acute thrombus in a deep vein. All VTE events were diagnosed using standard imaging techniques, including angiography, computed tomography angiography, ventilation-perfusion scan, or duplex ultrasonography. Deep surgical site infection (SSI) required a reoperation and was defined based on the Centers for Disease Control and Prevention criteria [25]. All study events were diagnosed and documented by the clinical treating team. All adverse events were reviewed by the Data and Safety Monitoring Board.

### Weighting of study outcomes

Outcome weights were derived for the study outcomes based on the results of a discrete choice experiment [23]. The experiment surveyed 232 orthopaedic trauma patients to quantify the relative importance of the included endpoints. Using the methods described by Udogwu et al [26], the relative importance of the component outcomes was used to calculate the outcome weights (Table 1) and determined a hierarchy of severity for the observed combination of events experienced during the first 90 days from injury. The weights of the component outcomes are reported relative to death, which has been weighted as 1.00.

**Table 1:** Weights for component outcomes derived from previously published data.

Component Outcome	Weight
Death	1.000
VTE Event (Pulmonary Embolism or Deep Vein Thrombosis)	0.055
Deep Surgical Site Infection	0.015
Bleeding Complication	0.010

### Statistical analysis

Previous research determined that patients preferred the oral administration of VTE prophylaxis over a subcutaneous injection, assuming the treatment benefits were similar [23]. We then compared the strength of the patients’ route preference with VTE outcome preferences relative to baseline risks. Based on the calculated margins, we determined that patients would be willing to accept a 9% increase in the probability of an adverse event to avoid VTE prophylaxis by skin injection. Therefore, a sample size of 160 patients in each treatment group would provide 80% power to detect a 59% treatment superiority, assuming a nonparametric distribution of the weighted outcome and a two-sided alpha of 0.05 [27].

All analyses followed the intention-to-treat principle. We used two techniques to compare the probability of treatment superiority of aspirin with LWMH—a Global Rank test and a weighted time to event analysis. For the Global Rank test, all study outcomes were multiplied by the calculated weight and summed for each patient [28]. Ranks were then assigned to patients so that an event free outcome receives a rank of one. All other combinations of outcomes experienced by patients in the study are ranked based on their cumulative weight. The ranks were compared using a Wilcoxon Rank Sums test. The treatment effect was reported as the Probability Index [29, 30], which is the probability that a randomly selected patient from the aspirin group has a superior outcome rank than a randomly selected patient from the LMWH group. A probability of 50% is considered a null effect.

**Fig 1:**
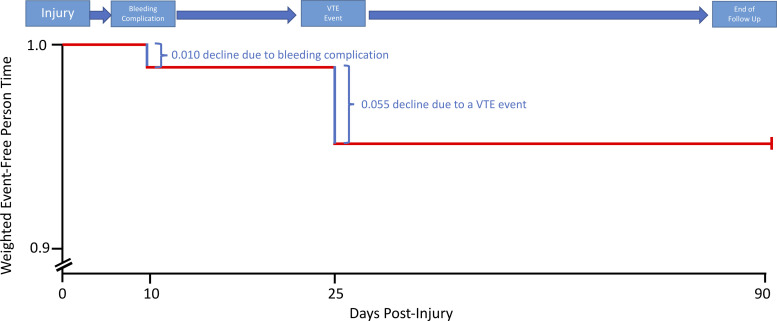
Schematic demonstrating how event-free person-time is weight based on the events experienced by an individual patient.

We also analyzed the data using a time to event model that weighted each event and then adjusted the patient’s health state based on that event for their remaining person-time [26]. Each patient was able to incur an unlimited number of events prior to 90-days post-injury or death, at which point they were censored in the analysis. All patients entered into the study event free. After an event, the remaining person-time that they contributed to the analysis was discounted at one minus the cumulative weight of the events the patient had experienced to that point in time (Fig 1). The treatment effect was reported as the probability of treatment superiority calculated as 1/(1-hazard ratio) [21]. The time to event analysis had 50% power to detect a 59% probability of treatment superiority with a two-sided alpha of 0.05 and the assumption that one-third of the patients would experience at least one study event within 90-days of injury. For patients with incomplete follow-up, last observation carry-forward was used to impute missing outcome weights [31]. We also performed an unweighted analysis comparing the study outcomes between the treatment groups using chi-square tests. The results of the unweighted analyses are available in the supporting information. All analyses were performed using R Version 3.6.1 (Vienna, Austria).

## Results

### Study population

**Fig 2:**
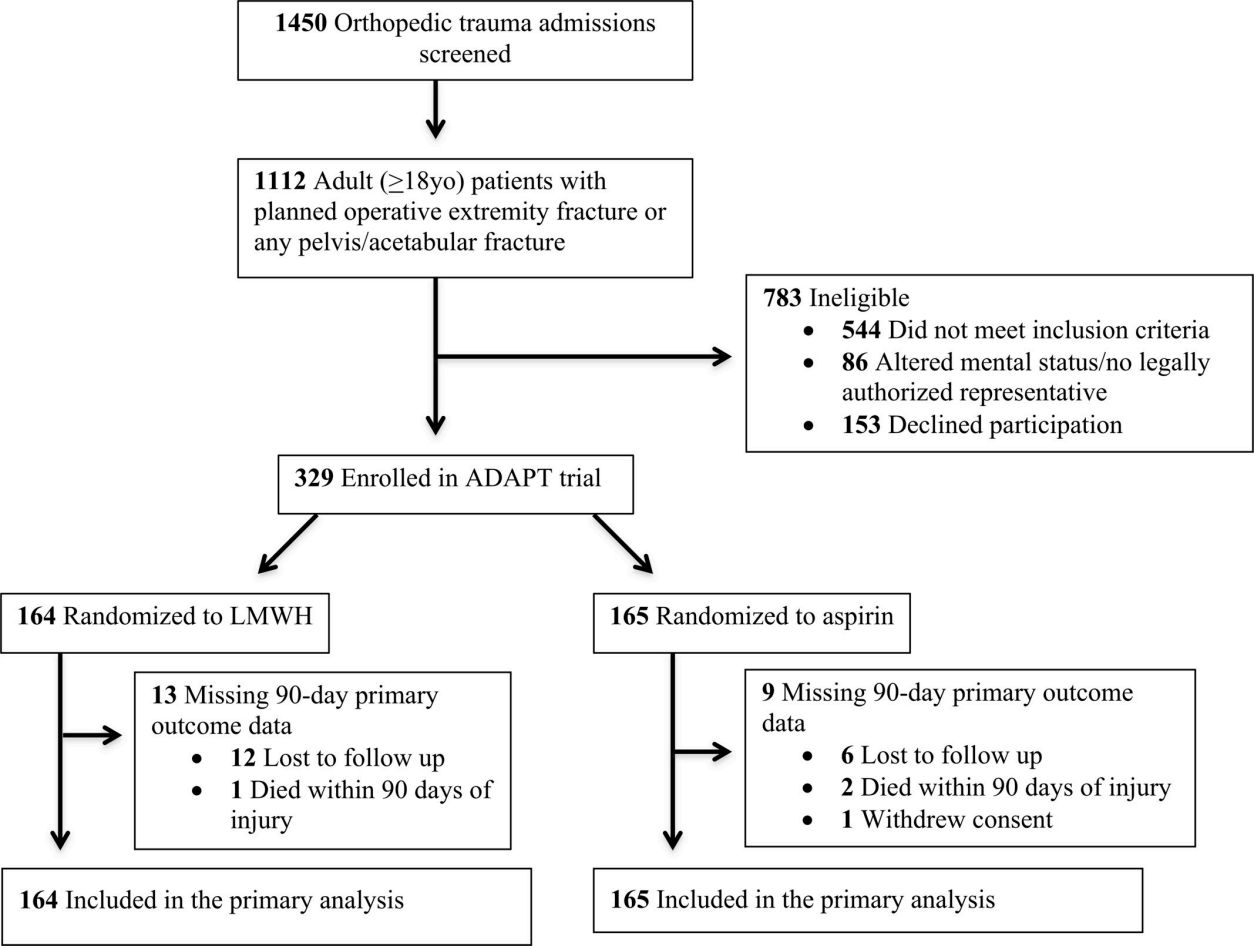
CONSORT diagram.

Of the 482 eligible patients, 329 were randomly assigned to receive VTE prophylaxis by LMWH or aspirin (LMWH, n = 164; aspirin, n = 165) (Fig 2). The mean age of the study participants was 47 years (SD, 20 years), and the majority (68%) were male. More than one-third of the participants had a recent history of tobacco use, 10% were diabetic, and 14% were taking daily aspirin before the fracture. Nearly 5% had a history of previous VTE. Most injuries were caused by blunt trauma (96%) to either the lower extremities or pelvis and acetabulum (92%). Nearly one-fourth of the fractures were open, and chest (25%) and head (22%) were common concomitant non-orthopaedic injuries. A full 90-day follow-up was achieved for 93% of the study population. Patient demographics by treatment arm are described in Table 2.

**Table 2:** Baseline patient characteristics (n = 329).

	LMWH (n = 164)	Aspirin (n = 165)
Age, mean (SD), yr	45.4 (20.4)	48.0 (18.6)
Sex, n (%)		
Male	119 (72.6)	104 (63.0)
Female	45 (27.4)	61 (37.0)
Race, n (%)		
White	97 (59.5)	106 (64.2)
Black	53 (3.3)	45 (27.3)
Hispanic	2 (1.2)	5 (3.0)
Mixed	6 (3.7)	3 (1.8)
Other	5 (3.1)	6 (3.6)
Current smoker, n (%)	62 (38.3)	65 (39.3)
History of venous thromboembolism, n (%)	7 (4.3)	8 (4.8)
Comorbidities, n (%)		
Peptic ulcer	3 (1.8)	12 (7.2)
Diabetes	16 (9.8)	17 (10.3)
Active cancer	2 (1.2)	5 (3.0)
Immunosuppression	9 (5.5)	8 (4.8)
Additional medications, n (%)		
Aspirin, daily pre-injury	23 (14.0)	22 (13.3)
Plavix, pre-injury	2 (1.2)	1 (0.6)
Oral contraceptive/estrogen	3 (1.8)	3 (1.8)
Body mass index, n (%), kg/m^2^		
Underweight (<18.5)	4 (2.5)	6 (3.7)
Normal weight (18.5–24.9)	57 (35.0)	57 (35.0)
Overweight (25.0–29.9)	53 (32.5)	47 (28.8)
Obese (≥30.0)	49 (30.1)	53 (32.5)
Injury Severity Score, mean (SD)	11.0 (5.7)	11.0 (6.6)
Mechanism of injury, n (%)		
Blunt	147 (94.2)	148 (97.4)
Penetrating	7 (4.5)	2 (1.3)
Other	2 (1.3)	2 (1.3)
Open fracture, n (%)	39 (23.4)	37 (22.4)
Fracture location, n (%)		
Upper extremity	41 (25.0)	42 (25.5)
Lower extremity and pelvis/acetabulum	149 (90.9)	154 (93.3)
Multi-limb	44 (27.0)	43 (26.1)
Non-orthopaedic injury (AIS ≥2), n (%)		
Abdomen	14 (8.5)	17 (10.3)
Head	36 (22.0)	35 (21.2)
Chest	43 (26.2)	39 (23.6)

LMWH, low molecular weight heparin; AIS, Abbreviated Injury Scale.

### Adherence with assigned intervention

The median time from admission to the initiation of study medication was one day in both treatment arms (aspirin IQR: 0–2; LMWH IQR: 0–1). The medication adherence for patients allocated to the aspirin arm (94.0%) was not significantly different than patients allocated to LWMH (91.1%, p = 0.18). Patients randomized to aspirin maintained daily prophylaxis significantly longer than those randomized to LMWH (median, 41 versus 26 doses; p<0.01).

Twenty-eight (17.0%) patients in the aspirin arm, and 57 (34.8%) patients in the LMWH arm, were administered prophylaxis only during their inpatient admission. Four (2.4%) patients in the aspirin arm and 3 (1.8%) patients in the LMWH arm were on prophylaxis for longer than 28 days.

### Global rank analysis

**Fig 3:**
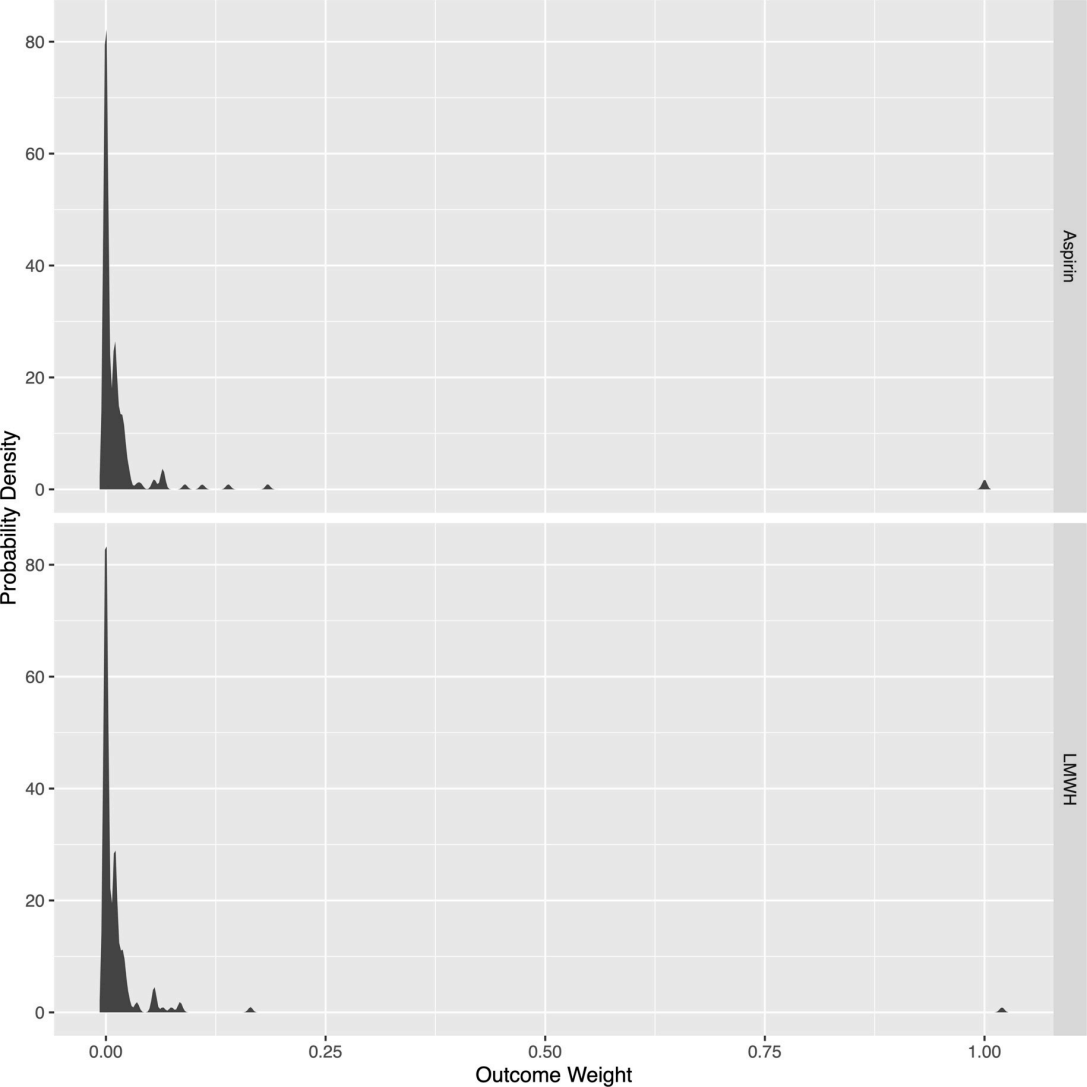
Probability density of weighted outcomes between treatment groups.

Overall, 18 different combinations of outcomes were experienced by patients in the study (Table 3). Ninety-nine patients in the aspirin group (59.9%) and 98 patients in the LMWH group (59.4%) were event-free within 90 days of injury. The cumulative weight of the events was 3.7 in the aspirin group and 2.5 in the LMWH group. Using a Global Rank test, the LMWH had a 50.4% (95% CI, 47.7–53.2%, p = 0.73) probability of treatment superiority over aspirin (Fig 3). Given the patient preference for aspirin [23], the probability of treatment superiority does not meet the 59% LMWH benefit threshold required for patients to change their preference to LMWH.

**Table 3:** Outcomes experienced by patients in the study and their assigned weight.

Rank	Weight	Overall N (%)	ASA N (%)	LMWH	Event Description
1	0	197 (59.9)	99 (60.3)	98 (59.4)	Event free
2	0.0099	61 (18.5)	32 (19.5)	29 (17.6)	Bleeding event
3	0.0153	16 (4.9)	7 (4.3)	9 (5.5)	Deep SSI
4	0.0198	21 (6.4)	10 (6.1)	11 (6.7)	Two bleeding events
5	0.0252	7 (2.1)	3 (1.8)	4 (2.4)	Deep SSI, bleeding event
6	0.0351	3 (0.9)	2 (1.2)	1 (0.6)	Deep SSI, two bleeding events
7	0.0398	1 (0.3)	0 (0.0)	1 (0.6)	Four bleeding events
8	0.0548	7 (2.1)	5 (3.1)	2 (1.2)	VTE
9	0.0647	5 (1.5)	1 (0.6)	4 (2.4)	VTE, bleeding event
10	0.0746	1 (0.3)	1 (0.6)	0 (0.0)	VTE, two bleeding events
11	0.0845	2 (0.6)	2 (1.2)	0 (0.0)	VTE, three bleeding events
12	0.0899	1 (0.3)	0 (0.0)	1 (0.6)	VTE, deep SSI, two bleeding events
13	0.1096	1 (0.3)	0 (0.0)	1 (0.6)	Two VTEs
14	0.1393	1 (0.3)	0 (0.0)	1 (0.6)	Two VTEs, three bleeding events
15	0.1644	1 (0.3)	1 (0.6)	0 (0.0)	Three VTEs
16	0.1842	1 (0.3)	0 (0.0)	1 (0.6)	Three VTEs, two bleeding events
17	1.000	2 (0.6)	0 (0.0)	2 (1.2)	Death
18	1.0198	1 (0.3)	1 (0.0)	0 (0.0)	Death, two bleeding events

### Time to event analysis

**Fig 4:**
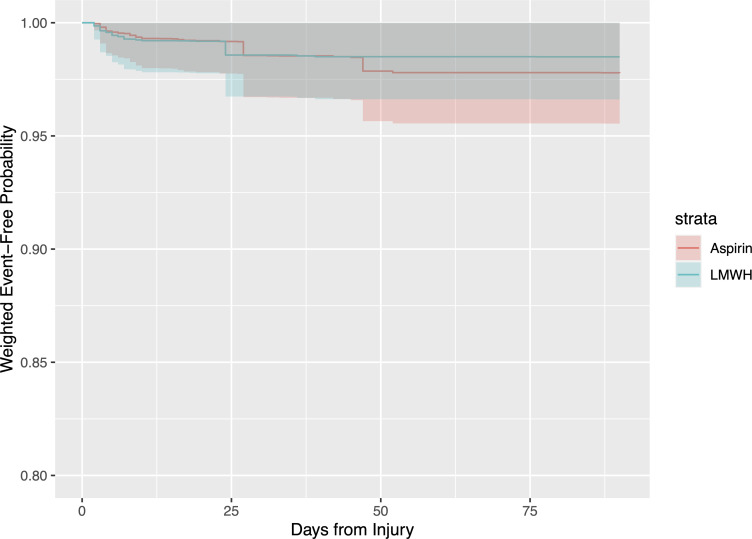
Weighted time to event analysis comparing the probability of being event-free within 90 days of injury. This analysis allows for multiple events for a given patient.

In the time to event analysis, the cumulative weighted probability of being event-free at 90-days post-fracture was 97.8% (95% CI, 95.5–1.00%) in the aspirin group and 98.5% (95% CI, 96.6–1.00%) in the LMWH group (Fig 4). LMWH had a 60.5% probability of treatment superiority over aspirin in this analysis but with considerable uncertainty (95% CI, 22.5–88.5%, p = 0.63) given the limited statistical power for this analysis.

## Discussion

In our primary comparison of aspirin with LMWH, there was no evidence of a significant difference in the probability of VTE prophylaxis superiority for orthopaedic trauma patients. In our time to event analysis, the point estimate for the VTE prophylaxis superiority of LMWH exceeded the 59% patient-important threshold. However, this finding was not statistically significant, given the considerable uncertainty around this point estimate.

The ADAPT trial is the first prospective randomized controlled trial to compare the effect of aspirin versus LMWH for VTE prophylaxis in orthopaedic trauma patients. We utilized patient preference data to weight and rank patient-important outcomes to determine the global VTE prophylactic benefit between the two medications [23]. Further, we considered the route of administration for the two medications and calculated a minimal importance threshold based on patients’ preference for oral administration over skin injections [23].

We aimed to describe medication differences under the totality of evidence gained from the trial. Under the ordinal rankings of the Global Rank test, the two medications were nearly indistinguishable in their overall performance. When time from injury was accounted for in the analysis, the probability of VTE prophylaxis superiority for LMWH increased after 45 days from injury, when several patients in the aspirin arm sustained subsequent events. Considering the longer median duration of VTE prophylaxis in the aspirin arm, the probability of VTE prophylaxis superiority may have diverged further if the duration of prophylaxis was consistent between the two arms.

Our analytic approach has several benefits. The composite outcome synthesizes the risk and benefits of VTE prophylaxis that clinicians and patients must consider when selecting a regimen. Further, the composite outcome included death, which then accounts for competing risks that often distort the interpretation of individual outcomes [21]. Allowing for multiple events for a given patients accounts for the correlation between events, and provides a more accurate representation of the patient experience.

This study had several limitations. The pragmatic nature resulted in some treatment contamination and a significant difference in the duration of prophylaxis. Study participants, clinicians, and research team members were not blinded to the treatment regimen. Lack of blinding and allocation concealment may have led to a treatment bias resulting in a differential threshold for diagnostic testing of study outcomes. However, we did not observe differential rates of diagnostic testing between the treatment groups. The study was a single-center study, which may limit its generalizability. The weights assigned to the study outcomes were derived from a VTE prophylaxis preference study. The bleeding complication weights did not account for the severity of bleeding events and more severe complications, such as intracranial bleeding or a retroperitoneal hematoma, that would likely have received a higher weight. The study had a broad eligibility criterion, and the VTE and bleeding risk may vary among this patient population. The study was adequately powered to detect a patient-important difference with the Global Rank test, but was limited in the time to event analysis. A larger prospective randomized study is needed to compare the VTE prophylactic effectiveness of these regimens for the specific outcomes included in our composite outcome and to determine the heterogeneity in treatment effect.

The findings of this randomized trial suggest that LMWH has a null to moderate global benefit for VTE prophylaxis in orthopaedic trauma patients. When considering patient preferences for an oral route of administration over a skin injection, aspirin is preferred based on the Global Rank analysis. The time to event framework suggests that the global VTE prevention benefits of LMWH may exceed the patient preference margin for aspirin. An evaluation of the effectiveness of these medications on specific clinical endpoints will require a considerably larger sample.

## Supporting information

S1 File CONSORT checklist. (DOC)

S2 File Study data. (XLSX)

S3 File Approved study protocol. (PDF)

S4 File Unweighted results. 90-day results using a intention to treat analysis.
(DOCX)
