# Supplementary material for: Aspirin versus low-molecular-weight heparin for venous thromboembolism prophylaxis in orthopaedic trauma patients: A patient-centered randomized controlled trial
Source: PLoS One. 2020 Aug 3;15(8):e0235628. doi: 10.1371/journal.pone.0235628 (PMC7398524; doi:10.1371/journal.pone.0235628)
Supplement: S3 File — (PDF) [file pone.0235628.s003.pdf]

Date: Wednesday, February 26, 2020 9:09:10 AM

Print Close

View: v2_Introduction Page

Introduction Page

1 * Abbreviated Title:
ADAPT

2 * Full Title:
A Different Approach To Preventing Thrombosis (ADAPT): a randomized-controlled trial comparing low molecular weight heparin to acetylsalicylic acid in orthopedic trauma patients.

3

* Select Type of Submission:

- (●) **IRB Application**
- ( ) Humanitarian Use Device (for FDA approved Indication & non-research purposes ONLY)
- ( ) Emergency Use
- ( ) Unsure if this proposal requires IRB review (Not Human Subject Research)

Note: The Type of Submission cannot be changed after this application has been submitted for review.

4 Original Version #:
1

ID: VIEW4DF8709A33C00
Name: v2_Introduction Page

View: v2_Research Team Information

Research Team Information

1 * Principal Investigator - Who is the PI for this study (person must have faculty status)? ***Faculty status is defined as being a full-time (>51% effort) faculty member holding one of the following titles at UM: Professor; Associate Professor; Assistant Professor.***
Deborah Stein

1.1 * Does the Principal Investigator have a potential conflict of interest, financial or otherwise, related to this research?
( ) Yes (●) **No**

2 Point of Contact - Who is the alternative point of contact for the PI? This person can be a study coordinator or any other study team member. In case the IRB cannot contact the PI, this person is a secondary person to contact:
Bryce Haac

2.1 Does the Point of Contact have a potential conflict of interest, financial or otherwise, related to this research?
( ) Yes (●) **No**

3 Other Team Members - list all additional members of the research team for this study. DO NOT include the PI or POC in this list:

Name	Edit Submission	cc on Email	Research Role	Has SFI?
Andrea Howe	yes	yes	Research Team Member	no

Name	Edit Submission	cc on Email	Research Role	Has SFI?
Gerard Slobogean	yes	yes	Research Team Member	no
Daniel Mascarenhas	yes	yes	Research Team Member	no
Christopher LeBrun	yes	yes	Other	no
Christopher Stewart	yes	yes	Research Team Member	no
Katherine Ordonio	yes	yes	Research Team Member	no
George Reahl	yes	yes	Research Team Member	no
Theodore Manson	yes	yes	Other	no
Daniel Connelly	yes	yes	Research Team Member	no
Richard Van Besien	no	no	Research Team Member	no
Nathan O'Hara	yes	yes	Research Team Member	no
Robert V. O'Toole	yes	yes	Other	no
Dimitrius Marinos	yes	yes	Research Team Member	no
Peter Berger	no	no	Research Team Member	no
Yasmin Degani	yes	yes	Research Team Member	no

IMPORTANT NOTE: All research team members (including PI) must have current CITI and HIPAA training completed.

ID: VIEW4DF85C16F2800
Name: v2_Research Team Information

View: v2_Resources

Resources

If this study is a collaborative UM/VA study, please clarify which resources are being used at each institution.

1 * Describe the time that the Principal Investigator will devote to conducting and completing the research:
Dr. Stein as the PI will devote ample time to conducting and completing the study.

2 * Describe the facilities where research procedures are conducted:
University of Maryland R. Adams Cowley Shock Trauma Center

3 * Describe the availability of medical and/or psychological resources that subjects might need as a result of anticipated consequences of the human research:
The patients enrolled in the study will be under the care of the Trauma and Orthopedic surgeons and they will have all of the R. Adams Cowley Shock Trauma Center medical resources available to them as part of the standard of care including the medical staff, imaging modalities, and pharmacologic treatment necessary to diagnose and treat any complication that should arise.

4 * Describe the process to ensure that all persons assisting with the research are adequately informed about the protocol, the research procedures, and their duties and functions:
All research team members will be trained on the protocol, the research procedures, and their duties and functions.

ID: VIEW4DF83CB976400
Name: v2_Resources

View: v2_Sites Where Research Activities Will Be Conducted

Sites Where Research Activities Will Be Conducted

1 * Is this study a:

- ○ Multi-Site
- ● **Single Site**

2 * Are you relying on an external IRB (not UM) to be the IRB of Record for this study?
○ Yes ● **No**

3 * Are any other institutions/organizations relying on UM to be the IRB of Record for this study?
○ Yes ● **No**

3.1 Attach the applicable regulatory documents here (i.e., IRB Authorization Agreement (IAA), FWA, local ethics approval, other IRB approvals, etc.). Final UM approval will be contingent upon final execution of all required regulatory approvals:

Name	Created	Modified Date
There are no items to display		

4 * Is UM the Coordinating Center for this study? (Applicable for multi-site studies. A Coordinating Center is responsible for overall data management, monitoring and communication among all sites, and general oversight of conduct of the project.)

- ○ Yes
- ● **No**

5 Is VA the Coordinating Center for this study? (Applicable for Collaborative studies between the VA, UM and other sites. A Coordinating Center is responsible for overall data management, monitoring and communication among all sites, and general oversight of conduct of the project)

- ○ Yes
- ○ No

6 * Institution(s) where the research activities will be performed:

- ☐ University of Maryland, The Founding Campus
- ☐ VAMHCS
- ☐ University of Maryland, Upper Chesapeake Kaufman Cancer Center
- ☐ UMB School of Medicine
- ☐ Marlene and Stewart Greenebaum Cancer Center
- ☐ University Physicians Inc.
- ☑ **Shock Trauma Center**
- ☐ General Clinical Research Center (GCRC)
- ☐ Maryland Psychiatric Research Center (MPRC)
- ☐ Johns Hopkins
- ☐ International Sites
- ☐ UMB Dental Clinics
- ☐ Center for Vaccine Development
- ☐ Community Mental Health Centers
- ☐ Private Practice in the State of Maryland
- ☐ Institute of Human Virology (IHV) Clinical Research Unit
- ☐ Joslin Center
- ☐ UMB Student Classrooms
- ☐ National Institute of Drug Abuse (NIDA)
- ☐ National Study Center for Trauma and EMS
- ☐ Univ of MD Cardiology Physicians at Westminster
- ☐ Nursing Homes in Maryland
- ☐ University of Maryland Biotechnology Institute
- ☐ Department of Health and Mental Hygiene (DHMH)
- ☐ Mount Washington Pediatric Hospital
- ☐ Capitol Region PG Hospital
- ☐ Maryland Proton Treatment Center
- ☐ Other Sites
- ☐ University of Maryland Medical System (Select below)

ID: VIEW4DF870DF2C000
Name: v2_Sites Where Research Activities Will Be Conducted

View: v2_Funding Information

Funding Information

1 * Indicate who is funding the study:

- [ ] Federal
- [ ] Industry
- [x] **Department / Division / Internal**
- [ ] Foundation
- [ ] Private
- [ ] State Agency

2 * What portion of the research is being funded? (Choose all that apply)

- [ ] Drug
- [ ] Device
- [x] **Staff**
- [ ] Participant Compensation
- [ ] Procedures
- [ ] Other

3 Please discuss any additional information regarding funding below:

ID: VIEW4DF85DF452400
Name: v2_Funding Information

View: v2_Research Protocol

Research Protocol

1 * Do you have a research protocol to upload?

- ( ) Yes
- (•) **No, I do not have a research protocol and will use the CICERO application to enter my study information**

2 If Yes, upload the research protocol:

Name	Created	Modified Date
There are no items to display		

ID: VIEW4E00563F8D000
Name: v2_Research Protocol

View: v2_Risk Level

Risk Level

What is the risk level of your study? (Ultimately, the IRB will determine the appropriate risk level and your designation is subject to change.)

* Choose One:

○ Minimal - The probability & magnitude of harm/discomfort anticipated in the research are not greater in and of themselves than those ordinarily encountered in daily life or during the performance of routine physical or psychological examinations/tests.

● **Greater Than Minimal - Does not meet the definition of Minimal Risk.**

ID: VIEW4E02805225800
Name: v2_Risk Level

View: v2_Type of Research

Type of Research

1 * Indicate **ALL** of the types of research procedures involved in this study (Choose all that apply):

- [x] **Use of unapproved drug(s)/biologic(s) or approved drug(s)/biologic(s) whose use is specified in the protocol.**
- [ ] Evaluation of food(s) or dietary supplement(s) to diagnose, cure, treat, or mitigate a disease or condition.
- [ ] Use of device(s) whose use is specified in the protocol
- [x] **Psychological/Behavioral/Educational Method or Procedure (i.e., survey, questionnaires, interviews, focus groups, educational tests).**
- [ ] Sample (Specimen) Collection and/or Analysis (including genetic analysis).
- [x] **Data Collection or Record Review (i.e., chart review, datasets, secondary data analysis).**
- [ ] None of the above.

2 * Is this study a clinical trial OR will this study be registered at ClinicalTrials.gov?
A clinical trial is a research study in which one or more human subjects are prospectively assigned to one or more interventions (which may include placebo or other control) to evaluate the effects of those interventions on health-related biomedical or behavioral outcomes.

● **Yes** ○ No

ID: VIEW4E0280569E000
Name: v2_Type of Research

View: v2_Lay Summary

Lay Summary

1 * Provide a summary of the background and purpose of the study in language that can be understood by a person without a medical degree.

Prevention of Venous thromboembolism (VTE) among high-risk trauma patients has been identified as one of the most important measures needed to improve outcomes for hospitalized patients by both the Agency for Healthcare Research and Quality and the Institute of Medicine. Known risk factors among these patients includes pelvic fractures, hip fractures, lower extremity fractures proximal to the knee, age greater than 30, head injury, prolonged immobilization, spinal cord injury and the need for blood transfusion. Over 60% of all injuries involve the musculoskeletal system, resulting in nearly $150 billion in health care costs across the United States. Limiting the impact of adverse outcomes associated with VTE for orthopaedic trauma patients in a cost effective manner represents an important opportunity to bolster the overall economic sustainability of a strained health care system.

Administration of prophylaxis following major orthopedic trauma has been proven to reduce illness and death from VTE. Historically, without prophylaxis the incidence of VTE was as high as 80% in this patient population, with a reduction to 1-11% using conventional pharmacological prophylactic strategies. Despite the release of numerous of VTE pharmacological prophylaxis guidelines for trauma patients, there has been significant discordance revolving around appropriateness of outcomes studied and the likelihood of adverse events. At present, the recommendations pertaining to orthopedic trauma patients are ambiguous at best, and largely extrapolated from arthroplasty and hip fracture trials in geriatric populations. Low Molecular Weight Heparin (LMWH) is endorsed, despite the potential increased risk in bleeding and associated wound complications such as hematoma and infection. Recently, several studies have examined the use of alternative pharmacologic agents such as acetylsalicylic acid (ASA) in hip and knee replacement, and have found it to be as effective as LMWH, while being associated with substantially fewer complications and costs. While these results are promising, they have yet to be widely adopted among orthopaedic trauma patients due to a current lack of evidence in the literature. A survey of Orthopedic Trauma Association (OTA) Member Practice Patterns and OTA Expert Panel Recommendations found a wide variability in practice patterns and poor scientific support for the various prophylaxis regimens highlighting the critical need for a standardized set of guidelines for VTE prophylaxis after orthopedic trauma to improve patient care. The purpose of this study is to perform a pragmatic randomized controlled trial to compare the use of low molecular weight heparin (LMWH) versus acetylsalicylic acid (ASA) for venous thromboembolism (VTE) prophylaxis in patients with high-risk fractures. It is our intention that this study will provide the needed evidence to determine the most effective agent for VTE prophylaxis with the lowest complication rate in high-risk orthopedic trauma patients.

ID: VIEW4E02805CF7000
Name: v2_Lay Summary

View: v2_Justification, Objective, & Research Design

Justification, Objective, & Research Design

If you uploaded a separate research protocol document in the 'Research Protocol' page, cite the applicable section and page numbers from that document in the answer boxes below.

1 * Describe the purpose, specific aims, or objectives of this research. State the hypothesis to be tested:

Purpose:
To perform a pragmatic randomized controlled trial of the use of low molecular weight heparin (LMWH) versus ASA for VTE prophylaxis in patients with high-risk fractures.

Specific Aims:
(1) To compare the bleeding and wound complication outcomes associated with LMWH (enoxaparin) versus ASA in patients receiving VTE prophylaxis following high-risk fractures.

(2) To compare the incidence of clinically important VTE events associated with LMWH (enoxaparin) versus ASA for VTE prophylaxis in patients receiving VTE prophylaxis following high-risk fractures.

(3) To compare the 6-month treatment costs associated with VTE prophylaxis using either LMWH or ASA for high-risk orthopedic trauma patients.

Hypothesis:
(1) Among high-risk orthopedic trauma patients receiving VTE prophylaxis, the rate of bleeding and wound complications will be lower for patients receiving ASA compared to those receiving LMWH.

(2) Among patients with high risk lower extremity fractures receiving VTE prophylaxis, the rate of VTE for patients receiving ASA will be no greater than those receiving LMWH.

(3) Among patients with high risk fractures receiving VTE prophylaxis, the 6-month treatment costs will be lower for patients receiving ASA compared to those receiving LMWH.

2 * Discuss the research design including but not limited to such issues as: probability of group assignment, potential for subject to be randomized to placebo group, use of control subjects, etc.:

Methods/Outcomes:
A randomized controlled trial will be conducted to assess the use of LMWH versus ASA for VTE prophylaxis in patients with high-risk fractures.
The aim-specific outcomes to be collected are as follows:
(1) A composite of the following major bleeding related and wound complications:
a. Fatal bleeding into a critical organ (retroperitoneal, intracranial, intraocular, intraspinal)
b. Clinically overt bleed with a > 2g/dL drop in Hb or requiring > 2U transfusion
c. Wound drainage or hematoma requiring reoperation
d. Diagnosis of deep surgical site infection
(2) VTE Events defined as a composite of any symptomatic proximal DVT (in the femoral or popliteal vessels), or PE (central, segmental or subsegmental). All VTE events will be radiologically confirmed.
(3) Cost of VTE prophylaxis treatment, VTE events and wound and bleeding related complications.

Data Collection:
Patients meeting inclusion/exclusion criteria will be prospectively randomized to one of two treatment arms. Block randomization with variable block sizes will be used. Patients will receive VTE prophylaxis as allocated, and followed for a 3month period for VTE events and bleeding and wound complications. Outcome data will be prospectively collected at followup appointments at 2 and 4 weeks +/- 2 days and 6 and 12 weeks +/- 1 week after discharge, and blind analysis and interpretation of results will be performed once 1/4 (n=240) of participants finish followup and again when one-half (n=480) of patients have completed 3-month followup for data safety monitoring. Additional monitoring will be performed if deemed necessary by the DSMB.

Data Analysis:
All data will be reported as mean and standard deviations for continuous variables and proportions and percentages for categorical data. Kaplan-Meier survival and Cox proportional hazard analysis will be completed for time to VTE and bleeding complication outcomes. Sub-group analysis will include Injury Severity Score and fracture location. A cost analysis will be conducted using a 6month and lifetime time horizon with a societal perspective. Component costs will consist of: VTE prophylaxis costs, unscheduled follow-ups, emergency room visit, hospital admission or unscheduled repeat surgical intervention for VTE or bleeding complications.

Study Population/Sample Size
Target Population:
Inclusion Criteria –
All patients 18 years old and older treated at a Level-1 Trauma center operatively or non-operatively for a pelvis or acetabulum fracture, or operatively for any extremity fracture proximal to the carpal or tarsal bones. Fractures may be open or closed, and patients may have other injuries or axial fractures.

Exclusion Criteria –
Patients receiving preexisting confounding prophylaxis or therapeutic anticoagulation (not to include anti-platelet agents) prior to admission or greater than two doses of confounding DVT prophylaxis at the study center since their injury.
Patients with preexisting coagulopathy
Patients with a previous history of VTE within the last 6 months
Patients deemed inappropriate for inclusion in this study by the treating physician
Patients who are pregnant
Patients with active bleeding precluding the use of anticoagulation
Impaired creatinine clearance <30ml/min at the time of randomization
History of Heparin Induced Thrombocytopenia or ASA or non-steroidal anti-inflammatory medication allergy
Patients who have an indication for therapeutic anticoagulation
Prisoners
NonEnglish Speaking patients.

Treatment Arms –
1: VTE prophylaxis with enoxaparin 30mg SC BID for patients with BMI of 30 or less, or enoxaparin 40mg SC BID for high BMI (>30) patients
2: VTE prophylaxis with ASA 81mg PO BID, an alternative standard of care regimen

Sample Size –
The primary outcome for this trial will be the incidence of major bleeding or wound complications. Based on a recent retrospective review at the R Adams Cowley Shock Trauma Centre of orthopedic trauma patients presenting over a 12-month period, we expect an overall incidence of our primary outcome to be 14.7% (95%CI 12.4%-17.1%) Using a 50% reduction rate in the incidence of bleeding and wound complications, 480 patients per treatment arm would be required to detect a difference (p=0.05, alpha=0.90, loss to followup=20%). Over a 12 month period (07/2013 – 06/2014), Shock Trauma Centre had 1204 patients present to the Trauma Resuscitation Unit with the orthopaedic injuries of interest. Seventy-three percent of patients met our inclusion/exclusion criteria (n=882). Using this data and assuming a 50% enrollment rate, the timeline for patient recruitment in the proposed trial will be 26 months.

3 * Describe the relevant prior experience and gaps in current knowledge. Describe any relevant preliminary data:

As discussed in the research design section, our research team has performed a retrospective review of all orthopedic trauma patients presenting over a 12 month period to the R Adams Cowley Shock Trauma Centre to determine the necessary sample size to detect a difference in the two treatment arms. The primary outcome for this trial will be the incidence of major bleeding or wound complications. Based on a recent retrospective review at the R Adams Cowley Shock Trauma Centre of orthopedic trauma patients presenting over a 12-month period, we expect an overall incidence of our primary outcome to be 14.7% (95%CI 12.4%-17.1%) Using a 50% reduction rate in the incidence of bleeding and wound complications, 480 patients per treatment arm would be required to detect a difference (p=0.05, alpha=0.90, loss to followup=20%). Over a 12 month period (07/2013 – 06/2014), Shock Trauma Centre had 1204 patients present to the Trauma Resuscitation Unit with the orthopaedic injuries of interest. Seventy-three percent of patients met our inclusion/exclusion criteria (n=882). Using this data and assuming a 50% enrollment rate, the timeline for patient recruitment in the proposed trial will be 26 months.

4 * Provide the scientific or scholarly background, rationale, and significance of the research and how it will add to existing knowledge:

Trauma is a well-described risk factor for the development of venous thromboembolism (VTE). Without any preventative measure, the reported incidence of VTE has historically ranged between 20 to 90%.(1) This variability is attributed to the heterogeneous patient population studied and the variety of methods used to make the

diagnosis of VTE. Geerts et al describe a cohort of 349 major trauma patients receiving no VTE prophylaxis that was prospectively followed and examined using bilateral contrast venography between 14 and 21 days following admission.(2) They found their cohort to have an incidence of deep vein thrombosis (DVT) of 58%, with 18% of these being proximal. Pulmonary Embolism had been described to occur in 2 to 22% of trauma patients, and is the third most common cause of death in trauma patients who survive the first 24 hours following an injury.(1-4) Identified risk factors for DVT and PE in trauma patients include age > 45 and 55 years respectively, bedrest >3 days, history of spine fractures, coma, quadra or parapalegia, pelvic fracture, lower extremity fracture, repair of a major lower extremity vein, complex open wounds and early blood transfusion.(2,5-7) These factors place orthopaedic trauma patients at a exceptionally high risk for VTE, particularly as the population continues to age and patients are able to survive severe injuries through coordinated acute trauma systems and improved care.(8)

Challenges exist in the assessment of VTE prophylaxis methods, especially in this patient population, and little in the way of high-level evidence is available. As such available guidelines have remained vague in their overall recommendations for patients with musculoskeletal injuries, and controversy exists as to the optimal methods of VTE prophylaxis.(9-11) An ideal agent would prevent VTE occurrence while minimize the risk of bleeding in this high-risk population.(8) Acetylsalicylic Acid (ASA, aspirin) is a widely used antiplatelet drug that may offer this ideal combination. It prevents platelet aggregation by inhibiting the production of thromboxane A2 by activated platelets. (12) Aspirin increases the bleeding time without affecting other coagulation parameters, and has been well established as an effective agent in the prevention of myocardial infarction and stroke.(13) Although limited information exists with respect to the ability of aspirin to prevent VTE events in orthopaedic trauma, there has been an accumulation basic science and clinical data supporting its overall effectiveness and safety in surgical patients.

The rational for use of aspirin in VTE prophylaxis is based on the evidence that arterial and venous thrombosis share a number of common features. Platelets, and fibrin are found in both arterial and venous thrombus, with a differing predominance of each component depending on the site and cause of thrombus formation.(14-21) There is evidence of both platelet activation and accumulation within venous thrombi, as well as increased levels of markers of platelet activation in patients with VTE.(14-18) P-selectin is one such protein exposed on the surface of activated platelets which functions to recruit leukocytes to the site of inflammation, and animal studies have shown that inhibition of this protein using aspirin can lead to the resolution of venous thrombosis without full anticoagulation.(19,20) Additionally, both arterial and venous thrombosis share common risk factors, and there is preliminary data showing that patients with spontaneous VTE are at an increased risk of adverse cardiovascular events.(21) This may provide an explanation for the overlapping efficacy of antiplatelet and anticoagulant agents in the treatment of both conditions.(21-23)

Clinically, a multitude of trials of variable size and quality have shown that antiplatelet drugs may have a protective effect against VTE. Earlier studies are best summarized in a systematic review from the Antiplatelet Trialists' Collaboration from 1994, which looked at 80 trials to determine the efficacy of antiplatelet therapy as prophylaxis against deep venous thrombosis or pulmonary embolism in surgical and high risk medical patients.(24) For DVT, They included data on 9623 patients (814 medical and 8809 surgical) from 53 randomized controlled trials, and reported an incidence of 640/2576 (24.8%) in antiplatelet drug recipients compared with 875/2605 (33.6%) in controls. This resulted in a 39% (SD 5%) odds reduction of DVT in favor of antiplatelet medication with the greatest benefit being seen in orthopedic patients as well high risk medical patients. Pulmonary embolism occurred in 47/ 4716 (1%) of patients allocated antiplatelet therapy compared with 129/4730 (2.7%) controls in 62 studies. This resulted in a 64% (SD 10%) reduction in odds of suffering a symptomatic pulmonary embolism in favor of antiplatelet medication.

To date, the Pulmonary Embolism Prevention (PEP) trial is the largest prospective randomized study comparing the effects of aspirin with placebo on the development of venous thrombosis in surgical patients, and was carried out between 1992 and 1998 on 17,444 patients across 5 countries.(25) 13,556 patients who required surgery for a hip fracture and 4088 patients who required elective hip arthroplasty were enrolled. All patients were randomized to receive aspirin 160 mg per day or placebo for 35 days. The criterion for a definite diagnosis of DVT and PE included the use of appropriate objective methods, and was limited to clinically important events as opposed to generalized screening for all events. Although some use this as a criticism of this trial, others feel that generalized screening leads to over-diagnosis of VTE, and can capture clinically asymptomatic and overall insignificant events.(26) The benefits of aspirin were evident in patients with hip fracture, where among 13,556 patients any VTE occurred in 105/6679 (1.6%) of patients assigned aspirin versus 165/6677 (2.5%) assigned placebo. This 36% relative reduction in VTE was significant (CI 17–50; p= 0.0003), and represented an absolute reduction in VTE of 9 per 1000 patients treated. Among elective arthroplasty patients, rates of venous thromboembolism were lower, but the proportional effects of aspirin were compatible to those patients with hip fracture. The authors reported no increase in severe bleeding events, including fatal bleeds and major hematomas. Therefore, the PEP study indicates that a 35 day course of aspirin reduces the incidence of clinically important venous thromboembolism in elective arthroplasty and hip fracture patients requiring surgery, compared to placebo controls.

With respect to head-to-head comparisons to other forms of chemo-prophylaxis, there have been several recent trials from the orthopaedic literature further supporting the use of aspirin for VTE prophylaxis in elective and limited trauma situations. Gehling et al randomized 287 patients with lower limb injuries who required casts to receive aspirin 500 mg bid or reviparin (Low-molecular weight heparin, LMWH) 1750 units daily. Symptomatic DVT proven by venography occurred in 7/144 (4.8%) of those assigned aspirin and 9/143 (6.3%) of those assigned LMWH, indicating that both modalities were similarly effective.(27) In two separate retrospective reviews, Bozic et al. as well as Vulcano et al. respectively reported the 30 and 90-day rates of clinically important VTE respectively in patients who underwent primary elective hip and knee replacement surgery with multimodal thromboprophylaxis.(28,29) Bozic and colleagues analyzed 93,840 patients who underwent primary total knee arthroplasty at 307 US hospitals over a 24-month period.(28)51,923 (55%) patients received warfarin, 37,198 (40%) received injectable agents, and 4719 (5%) received aspirin. The cohort from Vulcano and colleagues was smaller, however also assessed multiple modalities of VTE prophylaxis, with a total of 1,568 consecutive patients undergoing hip and knee replacement surgery, of whom 1,115 received aspirin, 426 received warfarin and 27 patients received low molecular weight heparin and warfarin.(29) Both studies showed that patients who received aspirin had lower odds for thromboembolism compared to warfarin patients but similar odds compared to injectable agents, including LMWH. Additionally there were no differences in risk of bleeding, infection, or mortality after adjustment for patient and hospital factors.(28,29) Similarly, Raphael et al reviewed 28,923 patients who underwent a total hip or knee arthroplasty over a 12 year period (2000-2012) receiving either aspirin (325 mg twice daily; 2800 patients) or warfarin prophylaxis (26,123 patients) and reported the incidence of symptomatic VTE, hematoma formation, infection, wound complications, and mortality up to 90 days postoperatively.(30) The overall symptomatic PE rate was lower ($p < 0.001$) in patients receiving aspirin (0.14%) than in the patients receiving warfarin (1.07%). The aspirin group also had significantly fewer symptomatic DVTs, wound-related problems and shorter hospital stays, all of which did not change with adjustment for patient and treatment factors.(30)

Obvious limitations and potential for bias exist with these retrospective reviews, however higher-level prospective trials offer similar results. In a 1996 publication, Lotke and colleagues randomized 388 patients to receive either aspirin (325mg bid) or warfarin (INR 1.2-1.5) following total hip or knee arthroplasty.(31) Patients were screened both pre and post-operatively using ventilation-perfusion scans an venography, and overall found to have no difference between groups with respect to incidence, size and location of DVT or PE. More recently another randomized trial compared 28 day extended postoperative VTE prophylaxis with aspirin (81mg daily) to LMWH (dalteparin 5000U SC daily) following hip arthroplasty and an initial 10-day period of LMWH prophylaxis.(32) The authors found that aspirin was noninferior ($P < 0.001$) to dalteparin and that clinically significant bleeding occurred in 5 patients (1.3%) receiving dalteparin and 2 (0.5%) receiving aspirin. The absolute between group difference in a composite of all VTE and clinically significant bleeding events was 1.7 percentage points (CI -0.3 to 3.8 percentage points; $P < 0.091$) in favor of aspirin. Furthermore, cost analysis modeling the use of aspirin against LMWH for arthroplasty patients found aspirin to be a cost-effective choice for prophylaxis, largely due to fewer bleeding complications as well as an overall lower incidence of VTE.

Overall, there is evidence that aspirin is an effective choice for VTE prophylaxis in elective orthopedic surgery patients and may decrease bleeding complications when compared to other chemo-prophylactic options. However, only limited data is available specific to orthopedic trauma patients, who may be at even higher risk for both VTE events and bleeding. Additionally, in an era of economic restraint, aspirin may offer additional benefits with respect to cost-containment through reduced upfront costs and associated complications compared to currently employed methods. With over 60% of all injuries involving the musculoskeletal system, resulting in nearly $150 billion in health care costs across the United States(33), limiting the impact of adverse outcomes associated with VTE for orthopedic trauma patients may represent an important opportunity to bolster the overall economic sustainability of a strained health care system, and further underscores the importance of future research in this area. We are proposing to conduct a pragmatic randomized controlled trial to evaluate ASA compared to LMWH for VTE prophylaxis in high-risk orthopedic trauma patients. If we are able to show in this trial that, as we hypothesize, ASA is as effective for preventing VTEs with a lower incidence of bleeding and infectious complication, this trial will provide the needed evidence to extend the use of ASA for VTE prophylaxis to the orthopedic trauma population with an added benefit of lowering the cost of patient care.

References

1. Shackford SR, Moser KM. Deep venous thrombosis and pulmonary embolism in trauma patients. J Intensive Care Med. 1988;
2. Geerts WH, Code KI, Jay RM, et al. A prospective study of venous thromboembolism after major trauma. N Engl J Med. 1994 Dec 15;331(24):1601–1606.
3. O'Malley KF, Ross SE. Pulmonary embolism in major trauma patients. The Journal of Trauma. 1990 Jun;30(6):748–750.
4. Sevitt S, Gallagher N. Venous thrombosis and pulmonary embolism. A clinico-pathological study in injured and burned patients. Br J Surg. 1961 Mar;48:475–489.
5. Shackford SR, Davis JW, Hollingsworth-Fridlund P, et al. Venous thromboembolism in patients with major trauma. Am. J. Surg. 1990 Apr;159(4):365–369.

6. Abelseth G, Buckley RE, Pineo GE, et al. Incidence of deep-vein thrombosis in patients with fractures of the lower extremity distal to the hip. J Orthop Trauma. 1996;10(4):230–235.
7. Rogers FB. Venous thromboembolism in trauma patients: a review. Surgery. 2001 Jul;130(1):1–12.
8. Hak DJ. Prevention of venous thromboembolism in trauma and long bone fractures. Curr Opin Pulm Med. 2001 Sep;7(5):338–343.
9. Falck-Ytter Y, Francis CW, Johanson NA, et al. Prevention of VTE in orthopedic surgery patients: Antithrombotic Therapy and Prevention of Thrombosis, 9th ed: American College of Chest Physicians Evidence-Based Clinical Practice Guidelines. Chest. 2012 Feb;141(2 Suppl):e278S–325S.
10. Hill J, Treasure T, National Clinical Guideline Centre for Acute and Chronic Conditions. Reducing the risk of venous thromboembolism in patients admitted to hospital: summary of NICE guidance. BMJ. 2010;340:c95.
11. Mont MA, Jacobs JJ, Boggio LN, et al. Preventing venous thromboembolic disease in patients undergoing elective hip and knee arthroplasty. J Am Acad Orthop Surg. 2011 Dec;19(12):768–776.
12. Catella-Lawson F, Reilly MP, Kapoor SC, et al. Cyclooxygenase inhibitors and the antiplatelet effects of aspirin. N Engl J Med. 2001 Dec 20;345(25):1809–1817.
13. Holbrook A, Schulman S, Witt DM, et al. Evidence-based management of anticoagulant therapy: Antithrombotic Therapy and Prevention of Thrombosis, 9th ed: American College of Chest Physicians Evidence-Based Clinical Practice Guidelines. Chest. 2012 Feb;141(2 Suppl):e152S–84S.
14. Sevitt S. The structure and growth of valve-pocket thrombi in femoral veins. J. Clin. Pathol. 1974 Jul;27(7):517–528.
15. Wang X, Hsu M-Y, Steinbacher TE, et al. Quantification of platelet composition in experimental venous thrombosis by real-time polymerase chain reaction. Thrombosis Research. 2007 Jan;119(5):593–600.
16. Schumacher WA, Heran CL. Effect of thromboxane receptor antagonists on venous thrombosis in rats. J Pharmacol Exp Ther. 2006 Jan 25;248(3):1109–1115.
17. Vesterqvist O, Gréen K, Johnsson H. Thromboxane and prostacyclin formation in patients with deep vein thrombosis. Thrombosis Research. 1987 Feb 15;45(4):393–402.
18. Klotz TA, Cohn LS, Zipser RD. Urinary excretion of thromboxane B2 in patients with venous thromboembolic disease. Chest. 1984 Mar;85(3):329–335.
19. Myers DD Jr, Rectenwald JE, Bedard PW, et al. Decreased venous thrombosis with an oral inhibitor of P selectin. Journal of Vascular Surgery. 2005 Aug;42(2):329–336.
20. Myers DD, Wrobleski SK, Longo C, et al. Resolution of venous thrombosis using a novel oral small-molecule inhibitor of P-selectin (PSI-697) without anticoagulation. Thromb Haemost. 2007 Mar 1;97(3):400–407.
21. Agnelli G, Becattini C. Venous thromboembolism and atherosclerosis: common denominators or different diseases? J. Thromb. Haemost. 2006 Sep 1;4(9):1886–1890.
22. Hurlen M, Abdelnoor M, Smith P, et al. Warfarin, aspirin, or both after myocardial infarction. N Engl J Med. 2002 Sep 26;347(13):969–974.
23. Schulman S, Lindmarker P, Holmström M, et al. Post-thrombotic syndrome, recurrence, and death 10 years after the first episode of venous thromboembolism treated with warfarin for 6 weeks or 6 months. J. Thromb. Haemost. 2006 Apr 1;4(4):734–742.
24. Trialists Coollaboration A. Collaborative overview of randomised trials of antiplatelet therapy--III: Reduction in venous thrombosis and pulmonary embolism by antiplatelet prophylaxis among surgical and medical patients. BMJ. 1994 Jan 22;308(6923):235–246.
25. Group PEPPTC. Prevention of pulmonary embolism and deep vein thrombosis with low dose aspirin: Pulmonary Embolism Prevention (PEP) trial. Lancet. 2000 Apr 15;355(9212):1295–1302.
26. Winters BS, Solarz M, Jacovides CL, et al. Overdiagnosis of Pulmonary Embolism: Evaluation of a Hypoxia Algorithm Designed to Avoid This Catastrophic Problem. Clin Orthop Relat Res. 2011 Oct 15;470(2):497–502.
27. Gehling H, Giannadakis K, Lefering R, et al. [Prospective randomized pilot study of ambulatory prevention of thromboembolism. 2 times 500 mg aspirin (ASS) vs. clivarin 1750 (NMH)]. Unfallchirurg. 1998 Jan 1;101(1):42–49.
28. Bozic KJ, Vail TP, Pekow PS, et al. Does aspirin have a role in venous thromboembolism prophylaxis in total knee arthroplasty patients? Journal of Arthroplasty. 2010 Oct 1;25(7):1053–1060.
29. Vulcano E, Gesell M, Esposito A, et al. Aspirin for elective hip and knee arthroplasty: a multimodal thromboprophylaxis protocol. International Orthopaedics. 2012 Jun 12;36(10):1995–2002.
30. Raphael IJ, Tischler EH, Huang R, et al. Aspirin: An Alternative for Pulmonary Embolism Prophylaxis After Arthroplasty? Clin Orthop Relat Res. 2013 Jul 2;472(2):482–488.
31. Lotke PA, Palevsky H, Keenan AM, et al. Aspirin and warfarin for thromboembolic disease after total joint arthroplasty. Clin Orthop Relat Res. 1996 Mar;(324):251–258.
32. Anderson DR, Dunbar MJ, Bohm ER, et al. Aspirin Versus Low-Molecular-Weight Heparin for Extended Venous Thromboembolism Prophylaxis After Total Hip ArthroplastyA Randomized Trial. Annals of internal medicine. 2013;158(11):800–806.
33. Haralson RH, Zuckerman JD. Prevalence, health care expenditures, and orthopedic surgery workforce for musculoskeletal conditions. JAMA. 2009 Oct 14;302(14):1586–1587.

ID: VIEW4E02805EA0C00
Name: v2_Justification, Objective, & Research Design

View: v2_Supporting Literature

Supporting Literature

1 * Provide a summary of current literature related to the research: ***If you uploaded a separate research protocol document in the 'Research Protocol' page, cite the applicable section and page numbers from that document in the answer box below.***

Please see the answer to #4. The scientific or scholarly background, rationale, and significance of the research in Justification, Objective and Research Design section for a detailed literature review related to the research and the citations.

2 If available, upload your applicable literature search:

Name	Created	Modified Date
1961 Br J Surg Sevitt.pdf	19/08/2015 11:43	19/08/2015 11:43
1974 J. Clin. Pathol. Sevitt.pdf	19/08/2015 11:43	19/08/2015 11:43
1977 Harris.pdf	19/08/2015 11:42	19/08/2015 11:42
1980 Br Med J McKenna.pdf	19/08/2015 11:42	19/08/2015 11:42
1984 Chest Klotz.pdf	19/08/2015 11:42	19/08/2015 11:42
1987 Thrombosis Research Vesterqvist.pdf	19/08/2015 11:41	19/08/2015 11:41
1988 J Intensive Care Med Shackford.pdf	19/08/2015 11:41	19/08/2015 11:41
1989 J Pharmacol Exp Ther Schumacher.pdf	19/08/2015 11:40	19/08/2015 11:40
1990 Am. J. Surg. Shackford.pdf	19/08/2015 11:40	19/08/2015 11:40
1990 The Journal of Trauma O'Malley.pdf	19/08/2015 11:40	19/08/2015 11:40
1992 The Journal of Trauma Knudson.pdf	19/08/2015 11:39	19/08/2015 11:39

Name	Created	Modified Date
1994 BMJ Trialists Coollaboration.pdf	19/08/2015 11:39	19/08/2015 11:39
1994 Clin Orthop Relat Res Fishmann.pdf	19/08/2015 11:37	19/08/2015 11:37
1996 N Engl J Med Geerts-1.pdf	19/08/2015 11:37	19/08/2015 11:37
1997 J Orthop Trauma Montgomery.pdf	19/08/2015 11:37	19/08/2015 11:37
1997 The Journal of Trauma Greenfield.pdf	19/08/2015 11:36	19/08/2015 11:36
1998 Unfallchirurg Gehling.pdf	19/08/2015 11:36	19/08/2015 11:36
2000 Lancet Group.pdf	19/08/2015 11:36	19/08/2015 11:36
2001 Curr Opin Pulm Med Hak.pdf	19/08/2015 11:36	19/08/2015 11:36
2001 N Engl J Med Catella-Lawson.pdf	19/08/2015 11:35	19/08/2015 11:35
2001 N Engl J Med Eriksson.pdf	19/08/2015 11:35	19/08/2015 11:35
2001 Surgery Rogers.pdf	19/08/2015 11:34	19/08/2015 11:34
2001 The Journal of Arthroplasty Brookenthal.pdf	19/08/2015 11:34	19/08/2015 11:34
2002 N Engl J Med Lassen.pdf	19/08/2015 11:34	19/08/2015 11:34
2002 The Journal of Trauma Rogers.pdf	19/08/2015 11:34	19/08/2015 11:34
2002 Thrombosis Research Jørgensen.pdf	19/08/2015 11:33	19/08/2015 11:33
2003 Circulation Anderson.pdf	19/08/2015 11:33	19/08/2015 11:33
2005 J Bone Joint Surg Br Steele.pdf	19/08/2015 11:32	19/08/2015 11:32
2005 Journal of Vascular Surgery Myers Jr.pdf	19/08/2015 11:31	19/08/2015 11:31
2006 J. Thromb. Haemost. Agnelli.pdf	19/08/2015 11:28	19/08/2015 11:28
2006 J. Thromb. Haemost. Schulman.pdf	19/08/2015 11:27	19/08/2015 11:27
2006 The Journal of bone and joint surgery American volume Stannard.pdf	19/08/2015 11:27	19/08/2015 11:27
2006 Thrombosis Research Isenbarger.pdf	19/08/2015 11:27	19/08/2015 11:27
2007 Acta Orthopaedica J Lapidus.pdf	19/08/2015 11:27	19/08/2015 11:27
2007 The Journal of Arthroplasty Burnett.pdf	19/08/2015 11:26	19/08/2015 11:26
2007 The Journal of Trauma Slavik.pdf	19/08/2015 11:26	19/08/2015 11:26
2007 Thromb Haemost Myers.pdf	19/08/2015 11:26	19/08/2015 11:26
2007 Thrombosis Research Wang.pdf	19/08/2015 11:25	19/08/2015 11:25
2008 Blood Rev. Watson.pdf	19/08/2015 11:25	19/08/2015 11:25
2008 The Journal of Arthroplasty Novicoff.pdf	19/08/2015 11:25	19/08/2015 11:25
2009 J Bone Joint Surg Br Goel.pdf	19/08/2015 11:24	19/08/2015 11:24
2009 J Orthop Trauma Slobogean.pdf	19/08/2015 11:24	19/08/2015 11:24
2010 National Quality Forum.pdf	19/08/2015 11:24	19/08/2015 11:24
2010 BMJ Hill.pdf	19/08/2015 11:24	19/08/2015 11:24
2010 Journal of Arthroplasty Bozic.pdf	19/08/2015 11:23	19/08/2015 11:23
2011 Arch Orthop Trauma Surg Hsu.pdf	19/08/2015 11:23	19/08/2015 11:23
2011 Circulation Mehran.pdf	19/08/2015 11:23	19/08/2015 11:23
2011 Clin Orthop Relat Res Winters.pdf	19/08/2015 11:22	19/08/2015 11:22
2011 J Am Acad Orthop Surg Mont.pdf	19/08/2015 11:22	19/08/2015 11:22
2012 Chest Falck-Ytter.pdf	19/08/2015 11:22	19/08/2015 11:22
2012 Chest Gould.pdf	19/08/2015 11:22	19/08/2015 11:22
2012 Chest Holbrook.pdf	19/08/2015 11:22	19/08/2015 11:22
2012 International Orthopaedics Vulcano.pdf	19/08/2015 11:21	19/08/2015 11:21
2012 J Am Acad Orthop Surg Tornetta.pdf	19/08/2015 11:21	19/08/2015 11:21
2012 J Orthop Surg (Hong Kong) Cossetto.pdf	19/08/2015 11:21	19/08/2015 11:21
2013 Anderson-1.pdf	19/08/2015 11:21	19/08/2015 11:21

Name	Created	Modified Date
2013 Ann Pharmacother Stewart.pdf	19/08/2015 11:19	19/08/2015 11:19
2013 BMJ Wiener.pdf	19/08/2015 11:19	19/08/2015 11:19
2013 Clin Orthop Relat Res Raphael.pdf	19/08/2015 11:19	19/08/2015 11:19
2013 J Orthop Trauma Sagi-1.pdf	19/08/2015 11:19	19/08/2015 11:19
2013 Journal of Trauma and Acute Care Surgery Brakenridge.pdf	19/08/2015 11:18	19/08/2015 11:18
2013 The Journal of Arthroplasty Mortazavi.pdf	19/08/2015 11:18	19/08/2015 11:18
2013 The Journal of bone and joint surgery American volume Lieberman.pdf	19/08/2015 11:18	19/08/2015 11:18
2013 The Journal of bone and joint surgery American volume Schousboe.pdf	19/08/2015 11:17	19/08/2015 11:17
2014 Barrack.pdf	19/08/2015 11:17	19/08/2015 11:17
2014 Am. J. Surg. Gritsiouk.pdf	19/08/2015 11:16	19/08/2015 11:16
2014 Orthopaedics & Traumatology Surgery & Research Parratte.pdf	19/08/2015 11:16	19/08/2015 11:16
2015 J Am Acad Orthop Surg Scolaro.pdf	19/08/2015 11:16	19/08/2015 11:16
2015 J Orthop Trauma Sagi.pdf	19/08/2015 11:15	19/08/2015 11:15
2015 J Orthop Trauma Selby.pdf	19/08/2015 11:15	19/08/2015 11:15
2015 Thrombosis Research Cohen.pdf	19/08/2015 11:15	19/08/2015 11:15

ID: VIEW4E02805A7E400
Name: v2_Supporting Literature

View: v2_Study Procedures

Study Procedures

If you uploaded a separate research protocol document in the 'Research Protocol' page, cite the applicable section and page numbers from that document in the answer boxes below. (If this study is a collaborative UM/VA study please list each procedure that is being conducted and the locations where it is being conducted.)

1 * Describe all procedures being performed for research purposes only (these procedures would not be done if individuals were not in the study) and when they are performed, including procedures being performed to monitor subjects for safety or to minimize risks:

All patients presenting to our center with orthopedic trauma will be screened for the study using the inclusion and exclusion criteria. If the patient is eligible, they will be asked to consent. Their care will not be affected should they refuse to participate in the study. There are no study specific procedures except for the followup survey that patients will be asked to complete at all of their followup appointments to evaluate for medication compliance and any diagnosis or treatment of VTE, PE or surgical complication since their last visit. Per standard of care, patients will be randomly assigned to take either ASA or LMWH prophylaxis for the recommended duration based on their injuries (up to 6 weeks). Patients will be followed while they are admitted to the hospital and at their followup appointments by the research team to collect data about their clinical course, but no tests or procedures will be performed solely for the purposes of this research. The only tests or procedures that are performed will be those that are medically indicated based on the clinical judgment of the patient's surgical team.

Patients on aspirin 81mg daily at time of admission will still be able to participate in this study since these patient still receive
VTE prophylaxis in addition to their home ASA in our current hospital protocol. Patients randomized to the ASA
group would have their ASA changed to our trial frequency of 81mg BID. If the patient requires a higher dose of
ASA that they take once daily, the patient will be excluded from the study to reduce confounding factors in final analysis. The patient's dose of home ASA will otherwise only be adjusted by the surgical team if indicated clinically. Patients randomized to the Lovenox group will receive both the lovenox prophylaxis and their home ASA as is current standard of care unless the clinical team decides to hold the ASA for reasons unrelated to the study.

2 * Describe all procedures already being performed for diagnostic or treatment purposes (if not applicable to the study, enter "N/A"):

Patients with signs or symptoms of venous thromboembolism will be worked up with the appropriate radiographic studies including but not limited to venous duplex ultrasound of the extremities or a CTA chest if clinically indicated as per standard of care. If a patient is diagnosed with a venous thromboembolism, treatment will be initiated per standard of care by the patient's primary team. If a patient has signs of a complication from chemoprophylaxis including bleeding or infection, management including diagnostic studies or laboratory tests and treatment will be conducted by the patient's primary team. No diagnostic workup or treatment of medical conditions will be initiated by the research team since this study is meant to be a pragmatic prospective randomized controlled trial. As such, patients who have no clinical indication for diagnostic workup or treatment will not have any additional testing or treatment for research purposes only.

All participants will be asked to wear pneumatic compression devices as an inpatient unless it is contraindicated based on their injury or their disease process. The decision to prescribe pneumatic compression devices will be made by the patient's clinical team as is current standard of care.

Followup of kidney function will be managed per the primary treating team as clinically indicated per current standard of care. Kidney function is tested regulary by the inpatient clinical team. If, from these routine tests, it is determined that the patient's kidney function requires them to be taken off the study prophylaxis treatment by the clinical team, the patient will be removed from the study. It is not routine for patients on these medications for outpatient prophylaxis to have their kidney function checked but the clinical team may choose to check function if there is cause for concern. The research team will not be involved in the decision of when or how to follow kidney function. No additional tests or follow-up will be required if a patient chooses to participate in the research study.

3 * Describe the duration of an individual participant's participation in the study:

Patients who consent to the study will receive VTE prophylaxis as allocated by block randomization with variable block sizes within 24 hours of admission to the hospital,

and they will be followed for a 3 month period for VTE events and bleeding complications. Outcome data will be prospectively collected at 2 and 4 weeks +/-2 days, and 6 and 12 +/- 1 week post injury either as an inpatient or at outpatient followup appointments. Patients may be called if they miss their appointments or the study team is unable to followup with them at their scheduled appointments for other reasons.

4 * Describe the amount of time it will take to complete the entire study:
The duration of the study will be the duration needed to enroll 960 participants and follow them for 3 months. Based on a retrospective review of shock trauma admissions we estimate that the study duration will require approximately 26 months to complete enrolment and an additional 3 months to complete followup for a total of 29 months.

5 * Describe any additional participant requirements:
None

ID: VIEW4E0280585B400
Name: v2_Study Procedures

View: v2_Sample Size and Data Analysis

Sample Size and Data Analysis

If you uploaded a separate research protocol document in the 'Research Protocol' page, cite the applicable section and page numbers from that document in the answer boxes below.

1 * Provide the rationale and sample size calculations for the proposed target population:
The primary outcome for this trial will be the incidence of major bleeding or wound complications. Based on a recent retrospective review at the R Adams Cowley Shock Trauma Centre of orthopedic trauma patients presenting over a 12-month period, we expect an overall incidence of our primary outcome to be 14.7% (95%CI 12.4%-17.1%) Using a 50% reduction rate in the incidence of bleeding and wound complications, 480 patients per treatment arm would be required to detect a difference (p=0.05, alpha=0.90, loss to followup=20%). Over a 12 month period (07/2013 – 06/2014), Shock Trauma Centre had 1204 patients present to the Trauma Resuscitation Unit with the orthopaedic injuries of interest. Seventy-three percent of patients met our inclusion/exclusion criteria (n=882). Using this data and assuming a 50% enrollment rate, the timeline for patient recruitment in the proposed trial will be 26 months.

2 * Provide the plan for data analysis. Include in the description the types of comparisons that are planned (e.g., comparison of means, comparison of proportions, regressions, analysis of variance, etc.), which is the primary comparison/analysis, and how the analyses proposed will relate to the primary purposes of the study:
All data will be reported as mean and standard deviations for continuous variables and proportions and percentages for categorical data. Kaplan-Meier survival and Cox proportional hazard analysis will be completed for time to VTE and bleeding complication outcomes. Sub-group analysis will include Injury Severity Score and fracture location. A cost analysis will be conducted using a 3 month and lifetime time horizon with a societal perspective. Component costs will consist of: VTE prophylaxis costs, unscheduled follow-ups, emergency room visit, hospital admission or unscheduled repeat surgical intervention for VTE or bleeding complications.

ID: VIEW4E02806052800
Name: v2_Sample Size and Data Analysis

View: v2_Sharing of Results

Sharing of Results

1 * Describe whether results (study results or individual subject results, such as results of investigational diagnostic tests, genetic tests, or incidental findings) will be shared with subjects or others (e.g., the subject's primary care physicians) and if so, describe how it will be shared:
The results of the overall study will not be shared with participants. The results will not affect the standard of care for each patient.

ID: VIEW4E02808CBD800
Name: v2_Sharing of Results

View: v2_Research with Drugs or Biologics

Research with Drugs or Biologics

You indicated on the "Type of Research" page that your study involves use of unapproved drug(s)/biologic(s) or approved drug(s)/biologic(s) whose use is specified in the protocol AND/OR evaluation of food(s) or dietary supplement(s) to diagnose, cure, treat, or mitigate a disease or condition.

1 * List all drugs/biologics to be administered in this study. Be sure to list each drug/biologic with its generic name only.

	Drug Name	FDA Approved	IND Number	PI IND Holder
View	Acetylsalacilic Acid	yes		
View	enoxaparin	yes		

2 * Attach the drug package insert or investigational drug brochure for the drugs being administered in this study:

Acetylsalacilic Acid (ASA) Prescribing Information.pdf	14/07/2015 16:26	14/07/2015 16:26
enoxaparin package insert.pdf	14/07/2015 16:23	14/07/2015 16:23

3 If more than one drug is administered, discuss the risk implications of drug/therapy interactions:
Patients will only receive either ASA or LMWH. They will not receive both drugs together so there will not be a risk of drug interaction between these two drugs. The only risk of drug interaction will be with other medications ordered by the patient's primary surgical team and decision to administer the drug based on known risk will be made by the primary team as it would be made whether the patient was a part of the study or not. If the primary team decides the risk is too great for the patient to receive the allotted chemoprophylaxis, the patient will be excluded from the study as described in participant requirements.

4 * Will you be using Investigational Drug Services?
○ Yes ● **No**

ID: VIEW4E0916E6E1400
Name: v2_Research with Drugs or Biologics

View: v2_Drug or Biologic Storage and Handling

Drug or Biologic Storage and Handling

4.1 * Do you have a plan regarding access controls for essential and approproate research personnel?
- [x] **Yes** ( ) No

4.2 * Will you have procedures for verifying physical access to the drugs(s)?
- [x] **Yes** ( ) No

4.3 * Will you label the drug(s) so that it is (they are) used appropriately for the study?
- [x] **Yes** ( ) No

4.4 * Will there be an establishment of a drug transfer process both into and out of the research site?
- [x] **Yes** ( ) No

4.5 * Will the storage of the drug(s) be in a secure environment and include locks on doors and controlled access?
- [x] **Yes** ( ) No

4.6 * Do you have a plan for only allowing trained personnel to administer the drug(s)?
- [x] **Yes** ( ) No

4.7 If applicable, will the storage of the drug(s) be at the appropriate temperature, with a storage and temperature log?
- [x] **Yes** ( ) No

ID: VIEW4E1D85CC57C00
Name: v2_Drug or Biologic Storage and Handling

View: v2_Placebos

Placebos

1 * Is this study placebo controlled?
- ( ) Yes [x] **No**

ID: VIEW4E0514EECCC00
Name: v2_Placebos

View: v2_Psychological/Behavioral/Educational Methods and Procedures

Psychological/Behavioral/Educational Methods & Procedures

You indicated on the "Type of Research" page that your study involves a psychological/behavioral/educational method or procedure such as a survey, questionnaire, interview, or focus group.

1 * Select all behavioral methods and procedures which apply to this study:
- [x] **Surveys/questionnaires**
- [ ] Key informant or semi-structured individual interviews
- [ ] Focus groups or semi-structured group discussions
- [ ] Audio or video recording/photographing
- [ ] Educational tests or normal educational practices (education instructional strategies, techniques, curricula, or classroom management methods)
- [ ] Individual or group behavioral observations
- [ ] Psychosocial or behavioral interventions
- [ ] Neuropsychological or psychophysiological testing
- [ ] Deception
- [ ] Other psychosocial or behavioral procedures

ID: VIEW4E09416F57800
Name: v2_Psychological/Behavioral/Educational Methods and Procedures

View: v2_Surveys/Questionnaires

Surveys/Questionnaires

You indicated that this study involves surveys and/or questionnaires.

If you uploaded a separate research protocol document in the 'Research Protocol' page, cite the applicable section and page numbers from that document in the answer boxes below.

1 * List all questionnaires/surveys to be used in the study, including both standardized and non-standardized assessments:
A questionnaire/case report form will be administered to patients at each followup appointment to evaluate for development of venous thromboembolism, bleeding or infectious complications treated by a physician since discharge, as well as compliance with prophylactic treatment at appointments up to 6 weeks postoperatively.

2 * Upload a copy of all questionnaires/surveys:

Name	Created	Modified Date
ADAPT Follow-up Questionnaire for Patients to Complete.doc	17/07/2015 15:14	15/02/2016 08:14

3 * What is the total length of time that each survey is expected to take?
5 minutes

4 * Are any of the questions likely to cause discomfort in participants or cause harm if their confidentiality were breached? (i.e., Illegal activities)
○ Yes ● **No**

5 * Do any questions elicit information related to the potential for harm to self or others?
○ Yes ● **No**

5.1 If Yes, what procedures are in place to assure safety?

ID: VIEW4E09460F5EC00
Name: v2_Surveys/Questionnaires

View: v2_Data Collection / Record Review

Data Collection/Record Review

You indicated on the "Type of Research" page that your study involves data collection or record review (i.e., chart review, not self-report).

1 * What type of data will be collected/analyzed in this study? (Check all that apply)
- [ ] Retrospective/Secondary Analysis (data has already been collected at the time of initial IRB submission)
- [x] **Prospective (data is not yet in existence and/or collected)**

2 * Will this study involve adding data to a registry or database for future use?
● **Yes** ○ No

3 * Will the data be released to anyone not listed as an investigator on the protocol?
○ Yes ● **No**

3.1 If Yes, give name(s) & affiliation(s):

ID: VIEW4E0E25A8CA400
Name: v2_Data Collection / Record Review

View: v2_Prospective Data

Prospective Data

You indicated that the study involves the collection of prospective data.

1 * Where is the data being collected from? (Check all that apply)
- [x] **Medical records**
- [x] **Medical images**
- [ ] Commercial (for profit) entity
- [ ] Publicly available records
- [ ] Schools
- [ ] Other

1.1 If Other, please specify:

2 * What data fields will you have access to/collect for the study? For example, name, initials, date of birth, Social Security number, income, demographic information, family units, housing, etc.
Name, MRN, date of birth, date of initial injury, date of admission, date of discharge, gender, height, weight, injuries, surgeries and dates, treating surgeon, laboratory studies, imaging studies, social history, pregnancy status, past medical history, injury severity score, medications administered and dates, transfusions administered, inpatient diagnoses

You can also upload a copy of the data fields/variables to be collected for the study:

Name	Created	Modified Date
There are no items to display		

ID: VIEW4E0E25B643800
Name: v2_Prospective Data

View: v2_Data Registry

Data Registry

You indicated that the study involves adding data to a registry or database for future use.

1 * What is the name of the registry/database to which data will be added? (If this study involves the VA, please state the name of the registry/repository and the CICERO protocol number it was approved under.)
CLOT database

2 * What is the purpose of the registry/database?
To record the information needed to evaluate the efficacy and safety of LMWH vs. ASA in one database that can be used for the analyses described in our specific aims.

3 * Who has oversight and controls access to the registry/database?
Dr. Bryce Haac will maintain oversight and control access to the database.

4 * Who will have access to the registry/database?
Only the researchers listed on the IRB and research staff.

5 * How long will the data be stored in the registry/database?
The data will be stored permanently in the registry.

6 * Are participants in the study allowed to request that their data be removed?
○ Yes ● **No**

6.1 If No, explain why subjects will not be able to request that their data be removed:
Participants can request to be removed from the study, but all data already collected will remain part of the registry to reduce the risk of bias in study results.

ID: VIEW4E0E25BCFA400
Name: v2_Data Registry

View: v2_Clinical Trial Registration

Clinical Trial Registration

You indicated on the "Type of Research" page that your study is a clinical trial.

1 * Does the UM Clinical Trials Registry policy require registration of this trial?
● **Yes** ○ No

2 * Has this trial been registered?
● **Yes** ○ No

ID: VIEW4E093BF078C00
Name: v2_Clinical Trial Registration

View: v2_Clinical Trial Registration Information

Clinical Trial Registration Information

You indicated that this clinical trial has been registered.

1 * Was this trial registered at www.clinicaltrials.gov?
● **Yes** ○ No

2 If no, was this trial registered on a site other than clinicaltrials.gov?
○ Yes ○ No

2.1 If Yes, specify the name of the other site:

2.2 Provide justification for registering this trial on this site:

3 * Registration Number
NCT02774265

ID: VIEW4E093BF1D0800
Name: v2_Clinical Trial Registration Information

View: v2_Participant Selection

Participant Selection

1 * How many local potential participants (or specimens/charts) do you anticipate will be screened for this study? ***Screening includes determining potential participants' initial eligibility for and/or interest in a study.***
2700

2 * How many participants (or specimens, or charts) will be enrolled/used for this study? ***A local prospective participant is considered enrolled in the study when a UM-approved Informed Consent Document (not including separate screening consent forms) is signed.***

Local - the number being enrolled at this site:
960

Worldwide - the number being enrolled total at all sites (including local enrollment):
960

3 * Gender:
- [x] **Male**
- [x] **Female**

4 * Age(s):
- [ ] 0 to 27 days (newborn infants)
- [ ] 28 days to 12 months (Infant)
- [ ] 13 months to 23 months (Toddler)
- [ ] 2 to 5 years (Preschool)
- [ ] 6 to 11 years (Child)
- [ ] 12 to 17 (Adolescents)
- [x] **18 years and older (Adult)**
- [ ] 89 years and older

5 * Race/Ethnicity:
- [x] **All Races Included**
- [ ] American Indian or Alaskan Native
- [ ] Asian/Other Asian
- [ ] Asian/Vietnamese
- [ ] Black or African American
- [ ] Hispanic or Latino
- [ ] Mixed Race or Ethnicity
- [ ] Native Hawaiian or Pacific Islander
- [ ] White or Caucasian

6

* Language(s):
- [x] **English**

- [ ] Chinese
- [ ] French
- [ ] Italian
- [ ] Japanese
- [ ] Korean
- [ ] Local Dialect
- [ ] Spanish
- [ ] Vietnamese
- [ ] Other

6.1 Specify Other:

7

* Are you excluding a specific population, sub-group, or class?

○ Yes ● **No**

7.1

If Yes, indicate your justification for excluding a specific population, sub-group, class, etc.:

ID: VIEW4E0E519C1D000
Name: v2_Participant Selection

View: v2_Vulnerable Populations

Vulnerable Populations

1 * Will you be targeting ANY of the following Vulnerable Populations for enrollment? (Select all that apply)

- [x] **Employees or Lab Personnel**
- [ ] Children (Minors)
- [x] **Cognitively Impaired/ Impaired Decision Making Capacity**
- [ ] Pregnant Women/Fetuses
- [ ] Wards of the State
- [x] **Students**
- [ ] Prisoners
- [ ] Nonviable Neonates or Neonates of Uncertain Viability
- [ ] Economically/Educationally Disadvantaged
- [ ] None of the above

Only select populations which you will be targeting for enrollment. Do not include populations that may be enrolled incidentally. Enrollment of a vulnerable population is considered to be “targeted” if the study team will be aware that a subject is from a vulnerable group as a result of interaction with the subject or collection of specific information about the subject, and the research team does not wish to exclude them. “Incidental” enrollment is limited to situations where a study team is unaware that a subject is from a vulnerable group.

ID: VIEW4E0E519917800
Name: v2_Vulnerable Populations

View: v2_Vulnerable Populations - Employees or Lab Personnel

Vulnerable Populations - Employees or Lab Personnel

You indicated that employees or lab personnel are included in this study.

1 * Describe how you will ensure particiption in this research will not affect employment and prevent undue influence:
Participation in this research is optional. Employment status will not be affected by the patient's decision on study participation nor by the data collected on the patient. The patient's direct employers will not have access to study information unless they are approved by the IRB to access this information as part of the research team.

ID: VIEW4E0E5192BA800
Name: v2_Vulnerable Populations - Employees or Lab Personnel

View: v2_Vulnerable Populations - Cognitively Impaired

Vulnerable Populations - Cognitively Impaired

You indicated that individuals who are cognitively impaired are included in this study.

1 * Describe how you will prevent undue coercion:
Patients who are cognitively impaired at the time of consent will only be included if the patient has a legally authorized representative (LAR) that can consent for him/her. This LAR is understood to be acting on the patient's behalf for healthcare decisions and will provide consent only if the LAR believes it is what the patient would have wanted.

2 * How will the capacity of these individuals to provide informed consent be assessed? How will you determine the need for a legally authorized representative?
The patient must be alert and oriented to consent. The attached evaluation to give consent form will be used to assess the patient's ability to comprehend the study and consent for him/herself. If the patient is unable to consent, a legally authorized representative will be allowed to consent. The LAR ID Form will be completed for patients for whom a LAR provides consent.

You can also upload a copy of the tool that will be used to evaluate capacity:

Name	Created	Modified Date
Evaluation to Give Consent.docx	24/08/2015 11:49	24/08/2015 11:49
LAR ID Form.pdf	24/08/2015 11:47	24/08/2015 11:47

3 * From which participants, who are not able to provide legally effective informed consent, will assent be obtained?

- ○ All participants
- ○ Some participants
- ● **None of the participants**

ID: VIEW4E0E51976DC00
Name: v2_Vulnerable Populations - Cognitively Impaired

View: v2_Vulnerable Populations - Students

Vulnerable Populations - Students

You indicated that students are included in this study.

1 * Describe the types of students that are included in this study:
Any student that is admitted to our center and meets study criteria will be able to participate in the study.

2 * Describe how you will prevent undue influence.
All of the student's PHI will remain confidential and no one with direct influence over the student's education will be given access to this information. If a member of the research team works directly with the student at their academic institution, that member will not participate in the consent and data collection on that patient.

ID: VIEW4E0E519F32000
Name: v2_Vulnerable Populations - Students

View: v2_Eligibility

Eligibility

1 * Do you have an existing Eligibility checklist(s) for this study?
○ Yes ● **No**

1.1 If Yes, upload here. If you need a template, you can download it by clicking **HERE**. The checklists you upload will also be available under the Documents tab of this application.

Name	Created	Modified Date
There are no items to display		

1.2 If No, create an eligibility checklist below:

List inclusion criteria (List each Inclusion Criteria individually, using the ADD button):

Number	Criteria

	Number	Criteria
View	1	All patients treated at a level-1 trauma center with any one or more of the following injuries: Pelvis fracture (operative or non-operative), Acetabulum fracture (operative or non-operative), or any operative extremity fracture (proximal to tarsals/carpals)
View	2	Age greater than or equal to 18 years old

List exclusion criteria (List each Exclusion Criteria individually, using the ADD button):

	Number	Criteria
View	1	Patients receiving pre-existing confounding prophylaxis or therapeutic anticoagulation (not to include anti-platelet agents) prior to admission or those receiving greater than two doses of LMWH since arrival to study center
View	2	Patients with pre-existing coagulopathy
View	3	Patients with a previous history of VTE within the last 6 months
View	4	Patients who are pregnant
View	9	Patients with active bleeding precluding the use of anticoagulation
View	12	Impaired creatinine clearance < 30ml/min at the time of randomization
View	13	History of Heparin Induced Thrombocytopenia or ASA allergy or non-steroidal anti-inflammatory medication allergy
View	14	Prisoners
View	15	Non-English Speaking patients.
View	16	Patients deemed inappropriate for inclusion in this study by their treating physician
View	17	Patients who have an indication for therapeutic anticoagulation
View	18	Patients who are not indicated for VTE prophylaxis based on their injury and expected hospital length of stay.
View	19	Patients currently taking a daily aspirin dose higher than 81mg daily

After entering the inclusion and exclusion criteria above, click the Save link. CICERO will automatically generate a printable Eligibility Checklist for you to use in your research. To review the checklist, click on the resulting link below. This checklist is also available under the Documents tab of this application.

Eligibility Checklist for HP-00065750_5 v5-9-2016-1462807851859(0.01)

ID: VIEW4E0E5185F9000
Name: v2_Eligibility

View: v2_Recruitment

Recruitment

1 * Describe plans for recruitment, including the identification of potential participants (or acquisition of charts/records/samples) and initial interactions with them: (If this study involves the VA please list all sites at which recruitment will take place.):

A list of all new orthopedic admissions will be provided to the research team daily by the orthopedic surgeons. The research team will then see all new admissions that meet the inclusion criteria and will determine study eligibility. If a patient is eligible they will be explained the purpose and components of the study and the patient will be enrolled in the study if they consent to participate.

2 * Describe measures that will be implemented to avoid participant coercion or undue influence (if not applicable to the study, enter "N/A"):

The research team will make it clear that participation is voluntary and that care will not be affected should a patient choose not to participate.

3 * Who will recruit participants (or acquire charts/records/samples) for this study? (Check all that apply)

- ☐ PI
- ☑ **Study Staff**
- ☐ Third Party

3.1 If you are using a third party, specify Third Party Recruiters:

4 Upload any recruitment tools such as screening/telephone scripts and introductory letters (do not upload advertisements here):

Name	Created	Modified Date
CLOT recruitment introductary statement.doc	20/07/2015 10:20	20/07/2015 10:20

ID: VIEW4E0BCAA0A6C00
Name: v2_Recruitment

View: v2_Advertising

Advertising

1 * Will you be using advertisements to recruit potential participants?
○ Yes ● **No**

ID: VIEW4E0BCCF811000
Name: v2_Advertising

View: v2_Research Related Risks

Research Related Risks

If you uploaded a separate research protocol document in the 'Research Protocol' page, cite the applicable section and page numbers from that document in the answer box below.

1 * Individually list each research-related risk, using a separate line for each. Next to each risk, delineate the likelihood/seriousness of the risk, and the provisions for minimizing the risk:

Potential risks to participating in this study are related to the potential side effects of the medications used for clot prevention in this study. These medications are both FDA approved and have an acceptable risk profile. Potential side effects for each treatment group include the following:

Treatment Group 1: Aspirin 81mg by mouth twice daily:
• Aspirin is given as a pill that is swallowed if a patient is able to swallow. If a patient is unable to swallow, this medication can be crushed and given through a feeding tube. There is a low risk that a patient may not absorb this medication completely if their gut is not working well.
• Allergic reactions can occur that range from itchy skin or hives to throat swelling and difficulty breathing. Patients who have an allergic reaction to aspirin will no longer be able to participate in the study, but information about their clinical course may still be collected.
• Bleeding, including wound hematoma (a collection of blood under the skin), may occur. The severity of these complications can vary. Bleeding complications can range from being clinically insignificant to requiring treatment with blood transfusion or an operation to control the bleed.
• Kidney Injury can occur from taking this medication or from other factors associated with injury. If a patient has acute kidney injury during their stay they may have to stop taking the study medication for clot prophylaxis as it may worsen kidney injury. Kidney function is tested regularly while patients are admitted to the hospital by their treating physicains. It is not routine for patients on these medications to have their kidney function checked after discharge from the hospital, but the clinical team may choose to check kidney function outpatient if there is cause for concern. The research team will not be involved in the decision of when or how to follow kidney function. If, from these tests, it is determined by the clinical team that a patient's kidney function requires them to be taken off the study prophylaxis treatment, they will be removed from the study but data may still be collected about their hospital course.

Treatment Group 2: Enoxaparin 30mg to 40mg given as an injection twice daily:
• Enoxaparin is given as an injection under the skin. Bruising can occur at the site of the injection. There is also a very low potential risk of infection at the site of injection.
• Allergic reactions can occur when taking Enoxaparin that are the same as those described in the Aspirin group. In addition, patients may have a reaction that causes their number of platelets, which help make blood clot, to drop called Heparin Induced Thrombocytopenia. If a patient experiences an allergic reaction to enoxaparin, the medication will be stopped and they will no longer be able to participate in the study, but information about their clinical course may still be collected.
• Bleeding complications as described in the aspirin group can also occur with enoxaprin and may require blood transfusion or an operation.
• Kidney Injury can also occur from taking this medication or from other factors associated with your injury. If a patient has acute kidney injury during their stay they may have to stop taking the study medication for clot prophylaxis as it may worsen kidney injury and at that point they will no longer be able to be participate in the study. Patients' kidney function will be monitored by their treating physicians as described above as is standard-of-care. The decision of whether to continue enoxaprin for prophylaxis will be up to the clinical team.

Despite potential risks, clot prophylaxis is standard of care in all hospitals in the U.S. for patients at high-risk of clot development because if a clot develops in the vessels in a patient's lungs (a pulmonary embolism) it can be fatal. In addition, the risk of wound complications is expected to be lower with aspirin than enoxaparin, the medication that all high-risk patients not in the study get. As such, there is not expected to be any additional risk to a patient from taking these medications that would not exist should a patient choose not to participate in the study.

In addition, all patients eligible for this study are at high-risk for developing a clot due to their fractures. While the medications we administer are used to prevent clots and reduce this risk, there is still a low risk that a patient may develop a blood clot despite preventative treatment. This risk exists even if a patient chooses not to participate in the study.

There are risks to taking both of these medications during pregnancy so pregnant patients cannot participate in the study. It is recommended that patients take measures to prevent pregnancy while on these medications. Should a patient become pregnant while taking the medications to prevent clot, they should notify their physicians who will decide whether they should continue to take the medication or not. They will also be withdrawn from the study but your clinical information may still be collected. The above risks exist whether or not a patient chooses to participate in the study.

There is also a risk for loss of confidentiality since personal information is collected for the study. This risk will be minimized by storing the data in a secure location such as a locked office or locked cabinet and electronic data will be password-protected. Only the research team will have access to this data. Study participants will never be identified individually in publications.

Finally there is potential that one of the questions that patients are asked by the research team at follow-up appointments will make them uncomfortable. The questions are not personal questions and so the risk of this is thought to be minimal. Should a question make a patient uncomfortable, they can decline to answer that question. These follow-up surveys will be short and should not require more than 5 minutes to complete and can be completed while patients wait for their appointment.

There may be risks in this study that are not yet known. We will conduct a safety analysis during the study and notify patients if there are any unforeseen risks that require us to end the study.

ID: VIEW4E1B52509F000
Name: v2_Research Related Risks

View: v2_Potential Benefits and Alternatives

Potential Benefits and Alternatives

If you uploaded a separate research protocol document in the 'Research Protocol' page, cite the applicable section and page numbers from that document in the answer boxes below.

1 * Describe the potential direct benefit(s) to participants:

As stated in the risks section, VTE and PE can be life threatening events and with chemical prophylaxis the incidence of these events is significantly reduced. In addition, based on the current literature it is hypothesized that ASA has a lower risk of bleeding and wound complications. Thus, if our hypothesis holds, the patients in the LMWH arm will have no greater risk of complications than patients who do not participate in the study and the patients in the ASA arm will benefit from a lower incidence of complications which translates to decreased transfusions, operations, and antibiotics. With fewer complications, length of stay is often shorter as well.

2 * Describe the importance of the knowledge expected to result from the study:
We will assess the efficacy and safety profile of products currently in use for VTE prophylaxis (enoxaparin/LMWH) and compare it to another commonly available medication (ASA), which may offer a decreased complication profile in association with lower costs while maintaining equal benefits in VTE/PE complication reduction. If ASA is shown to be non-inferior to LMWH, it will represent an important source of cost savings, as there is an 1800-fold difference in upfront material costs between the two treatment options. In addition, patient compliance may be higher with ASA as compared to LMWH given its easier route of administration and lower overall outpatient costs.

3 * Describe how the potential risks to participants are reasonable in relationship to the potential benefits:
As delineated in the potential risk to participants section, based on our hypothesis there should be no greater risk of VTE or PE in study patients compared to other high-risk orthopedic trauma patients who do not participate in the study. The current literature suggests that ASA is as effective as LMWH in VTE and PE prevention and is considered a standard of care alternative to LMWH. If anything, this literature suggests that ASA may have a lower complication rate in this patient population with respect to bleeding and wound complications. This can translate to fewer transfusions, operations, and antibiotics in addition to a potentially shorted hospital stay and lower health care costs.

4 * Describe the alternatives to participation in this study. If there are no alternatives, state that participation is voluntary and the alternative is not to participate. For intervention studies, describe appropriate alternative clinical procedures or courses of treatment available to subjects.
Participation in this study is voluntary and the alternative is not to participate.

ID: VIEW4E1B5251B0400
Name: v2_Potential Benefits and Alternatives

View: v2_Withdrawal of Participants

Withdrawal of Participants

If the questions below are not applicable to the research (i.e., chart review), enter "N/A".

1 * Describe anticipated circumstances under which subjects will be withdrawn from the research without their agreement:
Patients may be removed from the study without their agreement if they elope from the hospital, refuse their medications or are non-compliant with followup. Patient's who experience a new allergic reaction will no longer be able to receive the prophylactic medication and will need to be withdrawn, however their data may still be included in the analysis.

2 * Describe procedures for orderly termination:
If the patient can be contacted they will be informed of the termination and the reason for termination and will be reminded that their primary surgical team is still directing their clinical care. Their clinical care will not change as a result of their termination except as directed by the patient's surgical team as clinically indicated. No further data will be collected on these patients.

3 * Describe procedures that will be followed when subjects withdraw from the research, including partial withdrawal from procedures with continued data collection:
Patient's who withdraw their consent to participate at any time from the study will no longer have their data collected by the research team. Data collected prior to their withdraw, however, may still be included in data analysis. Clinical care will continue as directed per the patient's surgical team.

ID: VIEW4E1B52531F800
Name: v2_Withdrawal of Participants

View: v2_Privacy of Participants

Privacy of Participants

If the study does not involve interaction with participants, answer "N/A" to the questions below.

1 * Describe how you will ensure the privacy of potential participants throughout the study ***(privacy refers to persons and their interest in controlling access to themselves):***
Only patients who can consent themselves or have a LAR to consent will be included. Consent will be obtained without any additional non-hospital employees in the room except as allowed by the patient. Data collected and recorded about the patient will be done in a HIPAA-compliant manner and will be stored in a secure location with password protection. Data will be presented and published as aggregate data only in a manner that eliminates potential for individual identification.

2 * Describe the location where potential participants will receive research information and detail the specific actions the study team will take to ensure adequate privacy areas:
Research information will be relayed to patients in their hospital room with the same specific actions taken to protect privacy as is done when the primary surgical team presents HIPAA-protected information. No information will be shared with hospital employees not involved in the patient's care.

3 * Describe potential environmental stressors that may be associated with the research:
We do not foresee any potential environmental stressors associated with the research that are not related to the patient's clinical care. We feel all potential environmental stressors would be present regardless of participation in the study. However, if a patient feels discomfort with a specific question that they are asked related to the research they can opt out of answering that question.

4 * Will this study have a site based in the European Union?
○ Yes ○ No

5 * Will the study have planned recruitment or data collection from participants while they are located in the European Union?
○ Yes ○ No

Access link below for information about the EU General Data Protection Regulations to assist in answering these questions.

https://www.umaryland.edu/oac/general-data-protection-regulation/

ID: VIEW4E1B525B87C00
Name: v2_Privacy of Participants

View: v2_Confidentiality of Data

Confidentiality of Data

1 * Will stored research data contain identifiers or be able to be linked to and identify individual participants (either directly or through a code/research ID)?

- (selected) **Yes**
- ( ) No, the data will be stored de-identified/anonymous (stripped of all identifiers, no way to identify individual participants)

2 * Where will research data be kept (address electronic and paper data as applicable)? (If this is a VA study please list specific sites that data will be kept.)

All research data will be kept in a secure location on the University of Maryland Medical Center premises.

3 * How will such data be secured?

Electronic data will be password protected. Paper data will be kept in a locked file cabinet.

4 * Who will have access to research data?

Only members of the research team who have completed the necessary HIPAA training will have access to the research data.

5 * Will study data or test results be recorded in the participant’s medical records?

( ) Yes (selected) **No**

6 * Will any data be destroyed? ***(Please note that data for FDA regulated research and VA research cannot be deleted)***

( ) Yes (selected) **No**

6.1 If Yes, what data (e.g., all data, some recordings, interview notes), when and how?

7 Do you plan to obtain a Certificate of Confidentiality?

( ) Yes (selected) **No**

7.1 If Yes, upload your Certificate of Confidentiality. If you have not yet obtained the Certificate, please note that once it is obtained, you will need to submit an amendment to attach the document, make any needed changes to the submission and make needed changes to the Informed Consent Document.

Name	Created	Modified Date
There are no items to display		

8 * Discuss any other potential confidentiality issues related to this study:

There are no other potential confidentiality issues that our research team can foresee.

ID: VIEW4E1B5265E0400
Name: v2_Confidentiality of Data

View: v2_Monitoring Plan Selection

Monitoring Plan Selection

1 * Type of data safety monitoring plan for the study:

- ( ) Will use/defer to the external sponsor's Data Safety Monitoring Plan
- (selected) **Data Safety Monitoring by a Committee**
- ( ) Data Safety Monitoring by an Individual
- ( ) There is no data safety monitoring plan in place

ID: VIEW4E1B00E30D400
Name: v2_Monitoring Plan Selection

View: v2_Monitoring Plan - Committee

Monitoring Plan - Committee

You indicated that the monitoring will be done by a Committee.

1 * Will the Committee be Internal or External?

- (●) **Internal DSMB**
- ( ) External DSMB

2 * What data will be reviewed?

- [x] **Adverse Events**
- [x] **Enrollment Numbers**
- [x] **Patient Charts/Clinical Summaries**
- [x] **Laboratory Tests**
- [x] **Medical Compliance**
- [x] **Procedure Reports**
- [x] **Raw Data**
- [x] **Outcomes (Primary, Secondary)**
- [x] **Preliminary Analyses**
- [ ] Other

2.1 If Other, specify:

3 * What will be the frequency of the review?

- ( ) Annually
- ( ) Bi-Annually
- (●) **Other**

3.1 If Other, specify:
Review will be conduct at the after enrolment and followup is completed for 240 patients and 480 patients, and more frequently if deemed necessary by the DSMB.

4 * Safety monitoring results will be reported to:

- [x] **IRB**
- [ ] GCRC
- [ ] Sponsor
- [ ] Other

4.1 If Other, specify:

ID: VIEW4E1B025761800
Name: v2_Monitoring Plan - Committee

View: v2_Monitoring Plan - Internal DSMB

Monitoring Plan - Internal DSMB

You indicated that the monitoring committee will be an internal DSMB.

1 * List Internal DSMB Members:

	Name
View	James O'Connor, MD
View	Gordon Smith, PhD.

	Name
View	Saam Morshed, MD, PhD
View	Andrew Pollak, MD

2 * Confirm that no financial or other conflicts of interest exists for the above individuals.

- (●) **Yes** ( ) No

3 * Will there be an interim efficacy analysis?

- (●) **Yes** ( ) No

3.1 If Yes, when?

After enrolment and follow-up is completed for 240patients and 480patients.

4 * Briefly describe the DSM review process itself. Will it be an open or closed review to the investigator? Blinded/unblinded data? How will confidentiality of individual participant data be maintained?

The DSM review process will be a blinded data analysis that is closed to the investigator. All members of the DSMB have completed HIPAA training and will take the necessary steps to maintain confidentiality of individual participant data. In addition, prior to submission of the data to the DSMB, all patient identifiers that are not needed for midterm analysis will be removed from the database.

5 * What are the criteria defined in the protocol to be used for decision making regarding continuation, modification, or termination of study?

An independent, Data and Safety Monitoring Board (DSMB) composed of internal and external members will be responsible for monitoring the accumulated interim data as the trial progresses to ensure patient safety, review efficacy, evaluate recruitment, and assess overall data quality. At its first meeting the Data Safety and Monitoring Board (DSMB) will review definition of all outcomes and revisions to the protocol will be made as appropriate. As outlined above, several adverse events will be collected as primary and secondary outcomes of the study. The most severe of these events include:
- Fatal bleeding into a critical organ (retroperitoneal, intracranial, intraocular, intraspinal)
- Central pulmonary embolism (PE)
- Wound drainage or hematoma requiring reoperation

Summary data on these specific outcomes will be monitored by the DSMB when one-quarter of the patients (n=240) reach 3-month follow-up and again when one-half of patients (n=480) have completed 3-month follow-up, or more frequently if deemed necessary by the DSMB. These summaries will include analyses comparing rates of events by blinded treatment group, or in other subgroups requested by the DSMB. The analysis will be based on the alpha-spending function using an O'Brien-Flemming boundary. The pre-specified p-values for stopping the trial because of harms of with either treatment will be 0.000015 and 0.0031 at the first and second interim analyses, respectively. After each meeting, the DSMB will issue a written summary of its review of the study data, including adverse events, though these reports will not unblind the investigators as to potential treatment effects and/or study outcomes by treatment arm.

ID: VIEW4E1B0261D9400
Name: v2_Monitoring Plan - Internal DSMB

View: v2_Research Related Costs

Research-Related Costs

1 * Is the study's financial supporter (e.g., commercial sponsor, federal or state grant or contract, private foundation, physician-sponsor) covering any research-related costs?

- ( ) No
- (●) **Yes**

1.1 If Yes, check all that apply:

- [x] **Research-Related Services (personnel costs, tests, supplies, exams, x-rays, or consultations required in the study)**
- [ ] Investigational or Study Device
- [ ] Investigational or Study Drug
- [ ] Investigational Procedure(s)

1.2 If No, who is responsible for payment?

2 * Who is responsible for the uncovered research-related costs?

- [ ] Participant
- [ ] Sponsor
- [ ] UM
- [ ] Other
- [x] **There will be no uncovered research-related costs**

2.1 If Other, specify:

3 If the participant is responsible for any research-related costs, identify and estimate the dollar amount:

ID: VIEW4E1B5D9641800
Name: v2_Research Related Costs

View: v2_Compensation for Research-Related Injury

Compensation for Research-Related Injury

1 * Is this study under a master agreement that includes a provision requiring the sponsor to provide compensation to participants for research-related injury?
○ Yes ● **No**

1.1 If Yes, please provide the date and title of the agreement and upload the portion of the contract language relevant to compensation for research-related injury:

Name	Created	Modified Date
There are no items to display		

1.2 If No (the study is not under a master agreement), is there proposed contract language concerning payment to participants for treatment in the event of a research-related injury?
○ Yes ● **No**

1.2.1 If Yes, indicate the status of the contract review/approval with the ORD and upload the proposed language relevant to compensation for research-related injury:

1.2.2

Name	Created	Modified Date
There are no items to display		

ID: VIEW4E1B629EEC000
Name: v2_Compensation for Research-Related Injury

View: v2_Payment to Participants

Payment/Reimbursement to Participants

1 * Will participants receive payment (money, gift certificates, coupons, etc.) or reimbursement for their participation in this research?
○ Yes ● **No**

ID: VIEW4E1C52A5D7800
Name: v2_Payment to Participants

View: v2_HIPAA

HIPAA (Health Insurance Portability and Accountability Act)

1 * HIPAA applies to the University of Maryland School of Medicine, the University of Maryland School of Dentistry and the VA. Are you affiliated with, or will be accessing data from, any of these places? ● **Yes** ○ No

2 If Yes, will the study view, access, share, collect, use, or analyze health information that is individually identifiable under HIPAA? ● **Yes** ○ No

ID: VIEW4E1B0A2114400
Name: v2_HIPAA

View: v2_Protected Health Information

Protected Health Information (PHI)

You indicated that HIPAA applies and the study will view, access, share, collect, use, or analyze health information that is individually identifiable.

1 * Which PHI elements will be used or disclosed in this study? (Check all that apply)

- [x] **Name**
- [ ] Address (if more specific than Zip Code)

- [x] **Dates**
- [x] **Ages over age 89**
- [x] **Telephone numbers**
- [ ] Fax numbers
- [ ] Email addresses
- [ ] Social Security numbers
- [x] **Medical record numbers**
- [ ] Health plan beneficiary numbers
- [ ] Account numbers
- [ ] Certificate/license numbers
- [ ] Vehicle identifiers and serial numbers, including license plate numbers
- [ ] Device identifiers and serial numbers
- [ ] Web universal resource locators (URLs)
- [ ] Internet protocol (IP) address numbers
- [ ] Biometric identifiers, including fingerprints and voiceprints
- [ ] Full-face photographic images and any comparable images
- [ ] Any other unique identifying number, characteristic, or code, unless otherwise permitted by the Privacy Rule for re-identification
- [ ] None

2 * Why is the PHI necessary for this research?
If SSNs are going to be used, describe the specific use and type of SSN to be used (real, scrambled, last 4 digits).
PHI like date of birth is necessary to determine if there are any baseline differences in the two treatment groups. Medical record numbers and names will help us identify any duplicate injuries. Telephone numbers may be used to contact a patient if it is necessary to relay information to the patient about the study and they are not able to be seen in person.

3 * What is the source(s) of the PHI?
The hospital medical record, the treating surgical team and the patient

4 * Provide written assurance that Protected Health Information will not be reused. (Note: this refers to re-use on another study or for a purpose which has not been approved, not to the re-use of screening data during the current study).
The Protected Health Information will not be reused without IRB approval.

5 * How will permission to allow the use/disclosure of the individual's protected health information (PHI) be obtained? (Choose all that apply:)

- [x] **Obtain written authorization *(upload authorization form at the end of the application under "Consent and HIPAA Authorization Forms")***
- [x] **Requesting waiver/alteration of authorization (includes waiver of authorization for recruitment only)**
- [ ] Qualifies as a limited data set (LDS)

5.1 If you are using a limited data set (LDS), please attach the Data Use Agreement (DUA):

Name	Created	Modified Date
There are no items to display		

ID: VIEW4E1B0A24AA400
Name: v2_Protected Health Information

View: v2_Waiver/Alteration of Authorization

Waiver/Alteration of Authorization

You indicated that a waiver/alteration of authorization is requested.

1 * Provide rationale for how the research presents no more than minimal risk to the privacy of individuals:
The waiver of authorization will be for participant screening only. Only HIPAA trained research staff will be allowed to assess the PHI for screening purposes. All information used for screening will be kept confidential and will not be recorded outside of the medical record unless the patient agrees to participate in the study and signs a written HIPAA consent.

2 * Describe the plan to ensure the protection of PHI collected during this study from improper use and disclosure:
Only HIPAA trained research staff will be allowed to assess the PHI for screening purposes. All information used for screening will be kept confidential and will not be

recorded outside of the medical record unless the patient agrees to participate in the study and signs a written HIPAA consent. For study participants, data collected will be kept in a secure and locked location.

3 * Describe the plan to destroy the PHI collected during this study at the earliest opportunity consistent with the conduct of the research. If there is a need to retain PHI, provide a justification:
No PHI will be collected outside of the medical record on screened patients. Data including PHI will only be recorded in the study database after written consent is obtained from the patient. This data will not be destroyed. If any PHI is written down during the screening process it will be discarded in the appropriate bins on each hospital ward that are destined for shredding as per hospital protocol.

4 * Why could the research not practicably be done without access to and use of this PHI?
Screening of PHI is required to determine whether a patient is eligible for the study.

5 * Why could the research not practicably be done without the waiver or alteration?
Patient's cannot be identified for study participation without prior screening so a waiver is needed for this initial screening only.

6 * Will the subjects' PHI be disclosed to (or shared with) any individuals or entities outside of UM?
○ Yes ● **No**

6.1 If Yes, describe the individuals or entities outside of UM to whom PHI will be disclosed.

ID: VIEW4E1B0A2896400
Name: v2_Waiver/Alteration of Authorization

View: v2_Informed Consent Process

Informed Consent Process

If the study does not involve interaction with participants or a waiver of consent is being requested , answer "N/A" to the questions below.

1 * Indicate the type(s) of consent that will be involved in this study: (check all that apply)

- ☐ Not applicable (study may qualify as exempt)
- ☐ Request to Waive Consent/Parental Permission (Consent is not being obtained)
- ☐ Request to Alter Consent (Some Elements of Consent Waived)
- ☐ Request to Waive Documentation of Consent (Verbal/Oral Consent)
- ☑ **Written Consent Form**
- ☐ Electronic Consent

2 * Describe the Informed Consent process in detail:
Only patients who are able to consent themselves or have a LAR will be consented. The patient's must be alert and oriented and have a basic understanding of their clinical situation to consent for themselves. The patient will be given an opportunity to ask any questions after reading or being read the consent. Patient's will be screened for their understanding of consent with the standardized form that is provided in this IRB. After all questions are answered, if the patient agrees to participate in the study he/she will sign the consent form. If a patient is unable to consent for him/herself a legally-authorized representative may be identified to consent on the patient's behalf.

3 Confirm that the consent process will explain the following:

- The activities involve research.
- The procedures to be performed.
- That participation is voluntary.
- The name and contact information for the investigator.

* ● **Yes** ○ No

4 * Describe who will obtain Informed Consent:
The study coordinator and trained research team members will obtain informed consent.

5 * If obtaining consent from a legally authorized representative (LAR), describe how you will confirm that the individual is the LAR and can provide legally effective informed consent. *(Answer "N/A" if not obtaining consent from LARs)*
The standardized form that is included in the attachments for this IRB under the vulnerable populations section will be used to confirm the individual can legally provide effective informed consent for the patient.

6 * Describe the setting for consent:
The consent will be done within 24 hours of admission in the patients hospital room.

7 * Describe the provisions for assessing participant understanding:
The patient's must be alert and oriented and have a basic understanding of their clinical situation.The written consent can be read to the patient if requested. The patient will be given an opportunity to ask any questions. If there is any concern that participant understanding is not adequate they will be excluded from the study unless a LAR can be identified to consent. A standardized form will be used to verify the patient's understanding of the study and is attached in the vulnerable populations section of this IRB.

8 * Describe the consideration for ongoing consent:
Patient's will have an opportunity to withdraw from further study participation at each interaction with the research team. If the patient would like to withdraw at another time, they may contact the study coordinator to do so.

ID: VIEW4E1C661D0AC00
Name: v2_Informed Consent Process

View: v2_Consent Forms - Draft

Consent and HIPAA Authorization Forms - Draft

1 Upload all of your Consent Forms for approval. Use only Microsoft Word.

Name	Created	Modified Date
ADAPT Study Consent	28/07/2015 11:26	15/02/2016 07:38

IMPORTANT NOTE: the above list of consent forms (if any) are DRAFT versions. Under no circumstances should copies of these be distributed to patients/study subjects. If/when this research submission is approved by the IRB, approved consent forms will be available for download and use from the "Documents" tab of the Submission's workspace (click Exit and then look for the Documents tab - approved submissions only)

1A Archived Consent Forms:

Name	Created	Modified Date
There are no items to display		

2 Upload any HIPAA authorization forms here:

HIPAA consent for ADAPT	28/07/2015 11:27	14/01/2016 08:52

Please refer to HRPO's website for specific instructions for preparing informed consent documents and to access current templates:
http://hrpo.umaryland.edu/researchers/consents.html

ID: VIEW4E1C7712D3000
Name: v2_Consent Forms - Draft

View: v2_Organization Review Requirements (other than IRB)

Organization Review Requirements (other than IRB)

Answer the following questions to determine additional organizational review requirements:

1 **Department/Division Review** - All research submissions are required to undergo department/division/institutional review prior to IRB review. The following entity is listed as the required department/division/institutional review:

Trauma Rsrch-CORE

If this information is incorrect, please notify the HRPO office.

2 **RSC Review Criteria** - select 'Yes' if the answer is 'Yes' for any of the following questions. Review by the Radiation Safety Committee may be required.

* 2.1 Does the research involve the use of ionizing radiation? ○ Yes ● **No**

2.2 Does the research involve the sampling of radioactive human materials for subsequent use or analysis in a laboratory?

3 **IBC Review Criteria** - select 'Yes' if the answer is 'Yes' for any of the following questions. Review by the Institutional Biosafety Committee may be required.

* 3.1 Does the research involve human gene transfer?

-OR-
Does the research specifically apply to human studies in which induction or enhancement of an immune response to a vector-encoded microbial immunogen is the major goal, and such an immune response has been demonstrated in model systems, and the persistence of the vector-encoded immunogen is not expected? This type of research is often referred to as recombinant vaccine trials. ○ Yes ● No

3.2 Does the research involve the exposure of human subjects to pathogenic microorganisms, or the exposure of research staff to human subjects or samples known or reasonably expected to carry infectious disease(s)?

3.3 Does the research involve the sampling of materials from persons with no known infectious disease and where the only risk to study staff is occupational exposure to bloodborne pathogens as defined by the OSHA Bloodborne Pathogen Standard?

4 **Cancer Center Criteria** - Answer the following to determine if review by the Cancer Center (Hematology-Oncology) may be required.

* Does the protocol involve in any way studies related to the prevention, treatment, diagnosis, or imaging of neoplastic diseases? ○ Yes ● No

5 **General Clinical Research Center Review Criteria** - the GCRC offers free and/or cost shared resources for patient-oriented research. Click Here for more information.

Answer the following to determine if review by the GCRC may be required.

* Will the General Clinical Research Center (GCRC) facility or resources be used to conduct this activity? ○ Yes ● No

6 **VA Review Criteria** - Answer the following questions to determine if review by the VAMHCS R&D Committee may be required.

* 6.1 - Will the research be conducted by VA Investigators including PIs, Co-PIs, and Site Investigators on VA time (serving on compensated, WOC, or IPA appointments)? ○ Yes ● No

* 6.2 - Will the research utilize VA resources (e.g., equipment, funds, medical records, databases, tissues, etc.)? ○ Yes ● No

* 6.3 - Will the research be conducted on VA property, including space leased to and used by VA? ○ Yes ● No

PLEASE NOTE that the research may be funded by VA, by other sponsors, or may be unfunded.

ID: VIEW4E1AF91AB2400
Name: v2_Organization Review Requirements (other than IRB)

View: v2_Summary of Required Reviews (other than IRB)

Summary of Required Reviews (other than IRB)

1 **Additional Committee Reviews** - Based on your responses to the previous questions, you have identified the following additional reviews. To complete or view these additional committees' forms, click on the links below or exit this application and click on the appropriate button on left side of this submission's webpage.

Name of Related Submission

This protocol has no related submissions (RSC, GCRC, IBC, etc)

2 **Required Department and Specialty Reviews** - Based on the PI's organization (department, division, etc.) affiliation and answers to previous questions (use of Cancer Center, etc.), the organizations listed below are required to review this application. These reviews are conducted online and no additional forms or steps by the study team are required.

Name of Organization	**Review Status**
Trauma Rsrch-CORE	Complete
SOM Program in Trauma	Complete

ID: VIEW4E1C8D9AE4000
Name: v2_Summary of Required Reviews (other than IRB)

View: v2_Additional Documents

Additional Documents

1 Upload all additional documents here:

**Name**	**Created**	**Modified Date**
There are no items to display		

ID: VIEW4E0962513A000
Name: v2_Additional Documents

View: v2_Final Page of Application

Final Page of Application

You have reached the final page of this application. It is recommended that you click on the "Hide/Show Errors" link on the upper or lower breadcrumb row of this page. The "Hide/Show Errors" will do a search of your application, and highlight areas that are required or need to be completed prior to submitting.

By submitting this application, you are electronically routing the protocol for departmental scientific review and all other necessary reviews. According to information you have provided, this application will be routed to the following Departments for review prior to being forwarded to the IRB for review. These reviews are conducted online and no additional forms or steps by the study team are required.

Name of Organization	**Review Status**
Trauma Rsrch-CORE	Complete
SOM Program in Trauma	Complete

Required Safety Committee Reviews - In addition to the IRB, the following committees must review this submission. Each additional committee has a separate online form that the study team will be required to fill out. All committee applications (IRB plus those listed here) must be completed properly before the 'package' of applications can be submitted. The team may complete these additional forms in any order or at any time prior to submission of the IRB Application. To complete or view these additional committees' forms, click on the links below or exit this application and click on the appropriate button on left side of this submission's Workspace.

Name of Related Submission

This protocol has no related submissions (RSC, GCRC, IBC, etc)

You may check the progress of your application at any time by returning to the Workspace of this submission. A detailed history, including notes, dates, and times of events, is provided to you for this purpose.

If a reviewer returns the application to you, you must address their concerns and resubmit the protocol for review to all designated departments. After all departments have reviewed the application, it will automatically be sent to the IRB for review. Changes made to the submission after its approval must be submitted as modifications.

Investigator Attestation
By submitting this application, I, the Principal Investigator (PI), certify that the information provided in this application is complete and correct. Research will be conducted according to the submission as described, only by the approved principal investigator and study team members.

In addition, I agree to the responsibilities of a PI, including:

- Obtaining informed consent (if applicable) from all subjects as outlined in the submission.
- Reporting new information to the IRB per the requirements of the Investigator Manual.
- If Required, obtaining renewal of the protocol prior to the expiration of the approval period or halt all study activities upon study expiration.
- Accepting ultimate responsibility for the protection of the rights and welfare of human subjects, conduct of the study and the ethical performance of the project.
- Ensuring performance of all research activities by qualified personnel according to the IRB approved submission.
- Ensuring that research personnel have or will receive appropriate training.
- Ensuring no changes will be made in the research until approved by the IRB (except when necessary to eliminate apparent immediate hazards to subjects).

Click the "Finish" button and then click "Submit Application" in the submission Workspace.

ID: VIEW4E1B10C500000
Name: v2_Final Page of Application

View: IRB - Add a Team Member

Add a Team Member

1 * Select Team Member:
Andrea Howe

2 Research Role:
Research Team Member

3 * Edit Rights - Should this person be allowed full edit rights to the submission, including: editing the online forms and the ability to execute activities? Note - a person with edit rights will automatically be added to the CC list and will receive all emails regarding this protocol, even if the answer to #4 below is No.
(●) **Yes** ( ) No

4 * CC on Email Correspondence - Should this person be copied on all emails sent by the HRPO office to the PI/POC? Study team members with edit rights will automatically receive all emails:
(●) **Yes** ( ) No

5 * Does this study team member have a potential conflict of interest, financial or otherwise, related to this research?
( ) Yes (●) **No**

6 * Briefly describe experience conducting research and knowledge of the local study sites, culture, and society:
Andrea Howe is a clinical research coordinator with the department of Orthopaedics at University of Maryland

View: IRB - Add a Team Member

Add a Team Member

1 * Select Team Member:
Gerard Slobogean

2 Research Role:
Research Team Member

3 * Edit Rights - Should this person be allowed full edit rights to the submission, including: editing the online forms and the ability to execute activities? Note - a person with edit rights will automatically be added to the CC list and will receive all emails regarding this protocol, even if the answer to #4 below is No.
● **Yes** ○ No

4 * CC on Email Correspondence - Should this person be copied on all emails sent by the HRPO office to the PI/POC? Study team members with edit rights will automatically receive all emails:
● **Yes** ○ No

5 * Does this study team member have a potential conflict of interest, financial or otherwise, related to this research?
○ Yes ● **No**

6 * Briefly describe experience conducting research and knowledge of the local study sites, culture, and society:
Dr. Slobogean is an orthopedic trauma surgeon at Shock Trauma with significant clinical research experience.

View: IRB - Add a Team Member

Add a Team Member

1 * Select Team Member:
Daniel Mascarenhas

2 Research Role:
Research Team Member

3 * Edit Rights - Should this person be allowed full edit rights to the submission, including: editing the online forms and the ability to execute activities? Note - a person with edit rights will automatically be added to the CC list and will receive all emails regarding this protocol, even if the answer to #4 below is No.
● **Yes** ○ No

4 * CC on Email Correspondence - Should this person be copied on all emails sent by the HRPO office to the PI/POC? Study team members with edit rights will automatically receive all emails:
● **Yes** ○ No

5 * Does this study team member have a potential conflict of interest, financial or otherwise, related to this research?
○ Yes ● **No**

6 * Briefly describe experience conducting research and knowledge of the local study sites, culture, and society:
Experienced in conducting research and has good knowledge of the local study sites, culture, and society.

View: IRB - Add a Team Member

Add a Team Member

1 * Select Team Member:
Christopher LeBrun

2 Research Role:
Other

3 * Edit Rights - Should this person be allowed full edit rights to the submission, including: editing the online forms and the ability to execute activities? Note - a person with edit rights will automatically be added to the CC list and will receive all emails regarding this protocol, even if the answer to #4 below is No.
(●) **Yes** ( ) No

4 * CC on Email Correspondence - Should this person be copied on all emails sent by the HRPO office to the PI/POC? Study team members with edit rights will automatically receive all emails:
(●) **Yes** ( ) No

5 * Does this study team member have a potential conflict of interest, financial or otherwise, related to this research?
( ) Yes (●) **No**

6 * Briefly describe experience conducting research and knowledge of the local study sites, culture, and society:
Dr. Lebrun will be co-PI. He is an Air force/Orthopaedic traumatology expert. He has experience with frontline orthopaedic trauma care in a combat setting.

View: IRB - Add a Team Member

Add a Team Member

1 * Select Team Member:
Christopher Stewart

2 Research Role:
Research Team Member

3 * Edit Rights - Should this person be allowed full edit rights to the submission, including: editing the online forms and the ability to execute activities? Note - a person with edit rights will automatically be added to the CC list and will receive all emails regarding this protocol, even if the answer to #4 below is No.
(●) **Yes** ( ) No

4 * CC on Email Correspondence - Should this person be copied on all emails sent by the HRPO office to the PI/POC? Study team members with edit rights will automatically receive all emails:
(●) **Yes** ( ) No

5 * Does this study team member have a potential conflict of interest, financial or otherwise, related to this research?
( ) Yes (●) **No**

6 * Briefly describe experience conducting research and knowledge of the local study sites, culture, and society:
Experienced in conducting research and has good knowledge of the local study sites, culture, and society.

View: IRB - Add a Team Member

Add a Team Member

1 * Select Team Member:
Katherine Ordonio

2 Research Role:
Research Team Member

3 * Edit Rights - Should this person be allowed full edit rights to the submission, including: editing the online forms and the ability to execute activities? Note - a person with edit rights will automatically be added to the CC list and will receive all emails regarding this protocol, even if the answer to #4 below is No.
(●) **Yes** ( ) No

4 * CC on Email Correspondence - Should this person be copied on all emails sent by the HRPO office to the PI/POC? Study team members with edit rights will automatically receive all emails:
(●) **Yes** ( ) No

5 * Does this study team member have a potential conflict of interest, financial or otherwise, related to this research?
( ) Yes (●) **No**

6 * Briefly describe experience conducting research and knowledge of the local study sites, culture, and society:
Very experienced in conducting research and has good knowledge of local study sites, culture, and society.

View: IRB - Add a Team Member

Add a Team Member

1 * Select Team Member:
George Reahl

2 Research Role:
Research Team Member

3 * Edit Rights - Should this person be allowed full edit rights to the submission, including: editing the online forms and the ability to execute activities? Note - a person with edit rights will automatically be added to the CC list and will receive all emails regarding this protocol, even if the answer to #4 below is No.
(●) **Yes** ( ) No

4 * CC on Email Correspondence - Should this person be copied on all emails sent by the HRPO office to the PI/POC? Study team members with edit rights will automatically receive all emails:
(●) **Yes** ( ) No

5 * Does this study team member have a potential conflict of interest, financial or otherwise, related to this research?
( ) Yes (●) **No**

6 * Briefly describe experience conducting research and knowledge of the local study sites, culture, and society:
Very experienced in conducting research and has good knowledge of local study sites, culture, and society.

View: IRB - Add a Team Member

Add a Team Member

1 * Select Team Member:
Theodore Manson

2 Research Role:
Other

3 * Edit Rights - Should this person be allowed full edit rights to the submission, including: editing the online forms and the ability to execute activities? Note - a person with edit rights will automatically be added to the CC list and will receive all emails regarding this protocol, even if the answer to #4 below is No.
● **Yes** ○ No

4 * CC on Email Correspondence - Should this person be copied on all emails sent by the HRPO office to the PI/POC? Study team members with edit rights will automatically receive all emails:
● **Yes** ○ No

5 * Does this study team member have a potential conflict of interest, financial or otherwise, related to this research?
○ Yes ● **No**

6 * Briefly describe experience conducting research and knowledge of the local study sites, culture, and society:
Dr. Manson will be co-PI. He is an Orthopaedic Traumatology expert with an interest in orthopaedic anticoagulation research. He is an attending orthopaedic trauma surgeon at the R Adams Cowley Shock Trauma Center and cares for patients with complex fractures and multi-system trauma.

View: IRB - Add a Team Member

Add a Team Member

1 * Select Team Member:
Daniel Connelly

2 Research Role:
Research Team Member

3 * Edit Rights - Should this person be allowed full edit rights to the submission, including: editing the online forms and the ability to execute activities? Note - a person with edit rights will automatically be added to the CC list and will receive all emails regarding this protocol, even if the answer to #4 below is No.
● **Yes** ○ No

4 * CC on Email Correspondence - Should this person be copied on all emails sent by the HRPO office to the PI/POC? Study team members with edit rights will automatically receive all emails:
● **Yes** ○ No

5 * Does this study team member have a potential conflict of interest, financial or otherwise, related to this research?
○ Yes ● **No**

6 * Briefly describe experience conducting research and knowledge of the local study sites, culture, and society:
Experienced in conducting research and has good knowledge of the local study sites, culture, and society.

View: IRB - Add a Team Member

Add a Team Member

1 * Select Team Member:
Richard Van Besien

2 Research Role:
Research Team Member

3 * Edit Rights - Should this person be allowed full edit rights to the submission, including: editing the online forms and the ability to execute activities? Note - a person with edit rights will automatically be added to the CC list and will receive all emails regarding this protocol, even if the answer to #4 below is No.
○ Yes ● **No**

4 * CC on Email Correspondence - Should this person be copied on all emails sent by the HRPO office to the PI/POC? Study team members with edit rights will automatically receive all emails:
○ Yes ● **No**

5 * Does this study team member have a potential conflict of interest, financial or otherwise, related to this research?
○ Yes ● **No**

6 * Briefly describe experience conducting research and knowledge of the local study sites, culture, and society:
Richard Van Besien is a University of Maryland medical student who will be helping with the study as part of his research requirement for medical school.

View: IRB - Add a Team Member

Add a Team Member

1 * Select Team Member:
Nathan O'Hara

2 Research Role:
Research Team Member

3 * Edit Rights - Should this person be allowed full edit rights to the submission, including: editing the online forms and the ability to execute activities? Note - a person with edit rights will automatically be added to the CC list and will receive all emails regarding this protocol, even if the answer to #4 below is No.
● **Yes** ○ No

4 * CC on Email Correspondence - Should this person be copied on all emails sent by the HRPO office to the PI/POC? Study team members with edit rights will automatically receive all emails:
● **Yes** ○ No

5 * Does this study team member have a potential conflict of interest, financial or otherwise, related to this research?
○ Yes ● **No**

6 * Briefly describe experience conducting research and knowledge of the local study sites, culture, and society:
Very experienced in conducting research and has excellent knowledge of the local study sites, culture, and society.

View: IRB - Add a Team Member

Add a Team Member

1 * Select Team Member:
Robert V. O'Toole

2 Research Role:
Other

3 * Edit Rights - Should this person be allowed full edit rights to the submission, including: editing the online forms and the ability to execute activities? Note - a person with edit rights will automatically be added to the CC list and will receive all emails regarding this protocol, even if the answer to #4 below is No.
● **Yes** ○ No

4 * CC on Email Correspondence - Should this person be copied on all emails sent by the HRPO office to the PI/POC? Study team members with edit rights will automatically receive all emails:
● **Yes** ○ No

5 * Does this study team member have a potential conflict of interest, financial or otherwise, related to this research?
○ Yes ● **No**

6 * Briefly describe experience conducting research and knowledge of the local study sites, culture, and society:
Dr. O'Toole will be co-PI. He is Chief of Orthopaedics at the R Adams Cowley Shock Trauma Center with extensive experience in orthopaedic trauma clinical trials.

View: IRB - Add a Team Member

Add a Team Member

1 * Select Team Member:
Dimitrius Marinos

2 Research Role:
Research Team Member

3 * Edit Rights - Should this person be allowed full edit rights to the submission, including: editing the online forms and the ability to execute activities? Note - a person with edit rights will automatically be added to the CC list and will receive all emails regarding this protocol, even if the answer to #4 below is No.
● **Yes** ○ No

4 * CC on Email Correspondence - Should this person be copied on all emails sent by the HRPO office to the PI/POC? Study team members with edit rights will automatically receive all emails:
● **Yes** ○ No

5 * Does this study team member have a potential conflict of interest, financial or otherwise, related to this research?

○ Yes ● **No**

6 * Briefly describe experience conducting research and knowledge of the local study sites, culture, and society:
Experienced in conducting research and has good knowledge of the local study sites, culture, and society.

View: IRB - Add a Team Member

Add a Team Member

1 * Select Team Member:
Peter Berger

2 Research Role:
Research Team Member

3 * Edit Rights - Should this person be allowed full edit rights to the submission, including: editing the online forms and the ability to execute activities? Note - a person with edit rights will automatically be added to the CC list and will receive all emails regarding this protocol, even if the answer to #4 below is No.
○ Yes ● **No**

4 * CC on Email Correspondence - Should this person be copied on all emails sent by the HRPO office to the PI/POC? Study team members with edit rights will automatically receive all emails:
○ Yes ● **No**

5 * Does this study team member have a potential conflict of interest, financial or otherwise, related to this research?
○ Yes ● **No**

6 * Briefly describe experience conducting research and knowledge of the local study sites, culture, and society:
Peter Berger is a research Fellow with the University of Maryland Shock Trauma Orthopedic Department with experience conducting clinical trials for the department.

View: IRB - Add a Team Member

Add a Team Member

1 * Select Team Member:
Yasmin Degani

2 Research Role:
Research Team Member

3 * Edit Rights - Should this person be allowed full edit rights to the submission, including: editing the online forms and the ability to execute activities? Note - a person with edit rights will automatically be added to the CC list and will receive all emails regarding this protocol, even if the answer to #4 below is No.
● **Yes** ○ No

4 * CC on Email Correspondence - Should this person be copied on all emails sent by the HRPO office to the PI/POC? Study team members with edit rights will automatically receive all emails:
● **Yes** ○ No

5 * Does this study team member have a potential conflict of interest, financial or

otherwise, related to this research?

○ Yes ● **No**

6 * Briefly describe experience conducting research and knowledge of the local study sites, culture, and society:

Yasmin Degani, MPH is a clinical research specialist with the department of Orthopaedics at University of Maryland and has extensive experience coordinating orthopedic research and clinical trials.
