# Supplementary material for: Aspirin versus low-molecular-weight heparin for venous thromboembolism prophylaxis in orthopaedic trauma patients: A patient-centered randomized controlled trial
Source: PLoS One. 2020 Aug 3;15(8):e0235628. doi: 10.1371/journal.pone.0235628 (PMC7398524; doi:10.1371/journal.pone.0235628)
Supplement: S4 File — 90-day results using a intention to treat analysis. (DOCX) [file pone.0235628.s004.docx]

**S4 File.** 90-Day Results Using a Intention to Treat Analysis.

|  |  | **No. (%)** | | **Risk Difference, % (95% CI)** | **Relative Risk**  **(95% CI)** | ***P* Value** |
| --- | --- | --- | --- | --- | --- | --- |
|  |  | **Aspirin (n=165)** | **LMWH**  **(n=164)** |  |  |  |
| **Bleeding** | **Composite** | **53 (32.1)** | **52 (31.7)** | **0.4 (-9.6 – 10.4)** | **1.01 (0.74 – 1.39)** | **.94** |
|  | Transfusion | 39 (23.6) | 31 (18.9) | 4.7 (-4.1 – 13.5) | 1.26 (0.83 – 1.91) | .29 |
|  | Hemoglobin drop | 27 (16.4) | 37 (22.6) | -6.2 (-14.7 – 2.3) | 0.73 (0.47 – 1.14) | .16 |
|  | Hematoma | 1 (0.6) | 1 (0.6) | 0.0 (-2.3 – 2.3) | 0.99 (0.06 – 15.8) | >.99 |
|  | Gastrointestinal bleed | 1 (0.6) | 1 (0.6) | 0.0 (-2.3 – 2.3) | 0.99 (0.06 – 15.8) | >.99 |
|  | Other bleeding | 4 (2.4) | 0 (0) | 2.4 (0.1 – 4.8) | - | .04 |
|  |  |  |  |  |  |  |
| **Infection** | **Deep SSI^c^** | **15 (9.1)** | **12 (7.3)** | **1.8 (-4.3 – 7.8)** | **1.24 (0.60 – 2.57)** | **.56** |
|  |  |  |  |  |  |  |
| **DVT^b^** | **Composite** | **9 (5.5)** | **5 (3.0)** | **2.4 (-1.9 – 6.8)** | **1.79 (0.61 – 5.22)** | **.41** |
|  | Distal | 6 (3.6) | 3 (1.8) | 1.8 (-2.0 – 5.6) | 1.99 (0.51 – 7.81) | .32 |
|  | Proximal | 6 (3.6) | 3 (1.8) | 1.8 (-2.0 – 5.6) | 1.99 (0.51 – 7.81) | .32 |
|  |  |  |  |  |  |  |
| **PE^a^** | **Composite** | **2 (1.2)** | **6 (3.6)** | **-2.4 (-6.1 – 1.2)** | **0.33 (0.07 – 1.62)** | **.17** |
|  | Massive | 0 (0) | 0 (0) | - | - | - |
|  | Sub-massive | 0 (0) | 0 (0) | - | - | - |
|  | Symptomatic | 2 (1.2) | 6 (3.6) | -2.4 (-6.1 – 1.2) | 0.33 (0.07 – 1.62) | .17 |
|  |  |  |  |  |  |  |
| **Death** | **All-Cause** | **2 (1.2)** | **1 (0.6)** | **0.6 (-2.0 – 3.2)** | **1.99 (0.18 – 21.7)** | **0.56** |

1. PE= pulmonary embolism; b) DVT=deep vein thrombosis; c) SSI=surgical site infection
